# The Role of Global Appearance of Omnidirectional Images in Relative Distance and Orientation Retrieval

**DOI:** 10.3390/s21103327

**Published:** 2021-05-11

**Authors:** Vicente Román, Luis Payá, Adrián Peidró, Mónica Ballesta, Oscar Reinoso

**Affiliations:** Department of Systems Engineering and Automation, Miguel Hernández University, 03202 Alicante, Spain; lpaya@umh.es (L.P.); apeidro@umh.es (A.P.); m.ballesta@umh.es (M.B.); o.reinoso@umh.es (O.R.)

**Keywords:** omnidirectional imaging, global appearance description, localization, image retrieval, relative orientation, fourier signature, histogram of oriented gradients, gist

## Abstract

Over the last few years, mobile robotics has experienced a great development thanks to the wide variety of problems that can be solved with this technology. An autonomous mobile robot must be able to operate in a priori unknown environments, planning its trajectory and navigating to the required target points. With this aim, it is crucial solving the mapping and localization problems with accuracy and acceptable computational cost. The use of omnidirectional vision systems has emerged as a robust choice thanks to the big quantity of information they can extract from the environment. The images must be processed to obtain relevant information that permits solving robustly the mapping and localization problems. The classical frameworks to address this problem are based on the extraction, description and tracking of local features or landmarks. However, more recently, a new family of methods has emerged as a robust alternative in mobile robotics. It consists of describing each image as a whole, what leads to conceptually simpler algorithms. While methods based on local features have been extensively studied and compared in the literature, those based on global appearance still merit a deep study to uncover their performance. In this work, a comparative evaluation of six global-appearance description techniques in localization tasks is carried out, both in terms of accuracy and computational cost. Some sets of images captured in a real environment are used with this aim, including some typical phenomena such as changes in lighting conditions, visual aliasing, partial occlusions and noise.

## 1. Introduction

Nowadays, the presence of mobile robots has increased substantially in many areas, such as industry, households, transportation and education. As their abilities in perception and computation have increased, they have become an efficient tool to perform a wide range of tasks and they are expected to play a crucial role in the development of some activities. In this context, map building and localization are two of the main abilities a robot must develop to be really autonomous. Finding a solution to both problems, balancing accuracy, efficiency and robustness, is very important so that a robot can navigate autonomously and safely through real working environments [1].

In the field of perception, vision sensors have become a widespread tool to get information from the environment [2] due to several factors: the big amount of information they can capture with a relatively low cost; the availability of the data they provide (unlike GPS, whose signal may not be available temporarily, indoors or in narrow outdoor areas); the variety of configurations that they permit, from single-view cameras to binocular or trinocular systems; and the possibility of carrying out other high-level tasks such as people detection. Among the available configurations, catadioptric vision systems stand out thanks to their wide field of view, up to 360 deg around the camera axis [3]. The information captured with these systems can be projected onto varied surfaces, what permits different mathematical approaches depending on the type of task to solve [4]. Omnidirectional images are particularly effective comparing to conventional images due to the fact that they capture a global context of the environment. Therefore, with this kind of information, global features constitute an effective alternative, compared to local features, to many tasks, such as, for example, the reconstruction of complex indoor environments. In this regard, Sun et al. [5] and Pintone et al. [6] make use of deep learning approaches [7,8,9] to panoramic image analysis, with the objective of understanding the layout of indoor environments.

Solving the mapping and localization problems using only visual information is challenging. Images are highly dimensional data and they usually contain much redundant information. This information tends to change not only when the robot moves but also under some other usual circumstances such as changes in the external lighting conditions, noise during the acquisition of the image and occlusions due to the presence of, e.g., people in the environment. In addition, when a robot has to operate in indoor environments, it has to cope with the phenomenon of *visual aliasing*, which means that the visual information captured from very different positions may be very similar. Taking these facts into account, to build a functional visual model of the environment and to estimate the pose (position and orientation) of the robot within this model with robustness, it is necessary to find an alternative codification which is more efficient and robust against such phenomena.

Two main frameworks can be found in the literature to extract this information based either on local or on global appearance. The first family of methods consists in detecting some outstanding landmarks or regions and describing them using any algorithm that provides some invariance against transformations, such as SIFT [10], SURF [11], BRIEF [12], BRISK [13], ORB [14], FREAK [15] and LDB [16]. The second family consists of working with each scene as a whole, trying to build a unique descriptor per image that collects information on its global structure, using some approaches such as Principal Components Analysis [17], discrete Fourier transform [18], banks of Gabor filters [19], color histograms [20,21], directly subsampled versions of the original image [22] or Radon transform [23].

Traditionally, researchers have focused on the use of local appearance methods, and it can be considered a mature technology to solve the mapping and localization problems. Many approaches are proposed in the literature based on these descriptors [24,25,26,27,28]. Typically, they require the implementation of detection, description and tracking algorithms which tend to be relatively complex and computationally expensive. While they are often designed to be invariant against some movements of the robot, their behavior can deteriorate when other usual phenomena are present, such as changes in lighting conditions, occlusions, noise or visual aliasing. Some comparative analyses of this kind of descriptor can be found in [29,30]. Thanks to these comparatives, an optimal description method can be chosen and tuned depending on the environment and application.

Global-appearance approaches have been applied to these areas more scarcely. Since each image is described through a unique descriptor, they usually lead to models of the environment that can be handled intuitively by a human operator. The localization process is more straightforward, based on the pairwise comparison between descriptors. Some authors have made use of such approaches in the field of mobile robots, such as [31,32,33,34,35,36]. These techniques may be useful in unstructured environments where it is difficult to extract robust landmarks. As a drawback, they have been used typically to build topological models [37,38], since no metric information can be extracted from pure global appearance (unless additional sensory information is added).

In [39], a comparative evaluation of the performance of global-appearance methods in mapping tasks was carried out. However, we have not found any work in the literature that makes a deep and systematic study of the role of global appearance in localization tasks. Therefore, the objective of this paper is two-fold. On the one hand, we have chosen six widespread and accepted families of visual description methods, and we have adapted them to be used efficiently with omnidirectional visual information, in such a way that the resulting descriptors contain useful information to retrieve relative distance and orientation efficiently. To this aim, some algorithms have been implemented to estimate the relative position and orientation from these descriptors using purely visual information. On the other hand, we carry out a comparative evaluation of these descriptors in localization tasks and study their behavior against changes in the robot pose and other visual changes in the environment. Their relative performance has been tested and the influence of the most relevant parameters is assessed, completing the work presented in [39].

The remainder of the paper is structured as follows. Section 2 presents a state-of-the-art of global appearance description approaches and outlines the implementation of the three methods included in the evaluation. After that, in Section 3 the framework used to estimate the position and the orientation of the robot is detailed. Then, Section 4 presents the experimental setup and the set of images used in the experiments. The paper finishes with the results of the experiments, discussed in Section 5, and the conclusions and future lines of research in Section 6.

## 2. Global Appearance Descriptors

The objective of this section is two-fold. On the one hand, a state-of-the-art of global appearance descriptor is developed. On the other hand, a brief mathematical description of the methods included in the comparative analysis is made. Six families of global appearance methods have been chosen to be analyzed: methods based on the discrete Fourier transform (Section 2.1), on gradient orientation (Section 2.2), on the use of banks of Gabor filters (Section 2.3), on Speeded-Up Robust Features (SURF) description method (Section 2.4), on Binary Robust Independent Elementary Features (BRIEF) (Section 2.5) and on Radon transform (Section 2.6). A complete description of the methods can be found in [39,40,41]. However, for the sake of clarity, we have included an outline in this section.

We consider the movement of the robot is contained in the ground plane, and it captures images using an omnidirectional vision system mounted on its top. This system consists of a camera pointing towards a hyperbolic mirror, with their axes aligned and in vertical position. The complete experimental setup is presented in Section 4.

### 2.1. Descriptors Based on the Discrete Fourier Transform

The discrete Fourier transform (DFT) has been used by many researchers to extract the most relevant information from scenes. For example, Oliva and Torralba [19] propose using a windowed 2D Fourier transform, that permits defining some circular windows to select spatial information around some specific pixels in the scene. Ishiguro and Tsuji [42] propose an alternative approach, named Fourier Signature (FS), which is designed to be used on panoramic images. Menegatti et al. showed the robustness of this representation to build a model of an environment and to estimate the position of a vehicle using a Monte Carlo approach [18,31], in a relatively small environments and controlled conditions. Stürzl et al. [43] propose a visual homing algorithm based on the Fourier Signature, but the panoramic scene is previously reduced to a unidimensional array. Horst and Möller use it in visual place recognition [44].

The Fourier Signature (FS) permits obtaining a descriptor which is invariant against rotations of the robot in the ground plane when using panoramic images. For this reason, this is the DFT-based representation we have chosen in this comparative evaluation. The description process starts from a panoramic scene $f\left(x,y\right)\in R^{N_{1}\times N_{2}}$. Initially, the image can be subsampled to obtain a lower number of rows $k_{1}<N_{1}$ ($k_{1}=1$ in [43]). The FS of the resulting scene $f\left(x,y\right)\in R^{k_{1}\times N_{2}}$ is the matrix $F\left(u,y\right)\in C^{k_{1}\times N_{2}}$ obtained after calculating the unidimensional DFT of each row of the image. In the frequency domain, the main information is concentrated in the low frequency components, and the high frequency components tend to be more contaminated by the possible presence of noise in the original image. Taking this fact into account, by retaining the $k_{2}$ first columns and discarding the remainder, a compression effect is achieved. The new complex matrix, with $k_{1}$ rows and $k_{2}$ columns, can be expressed as a magnitudes matrix A(u,y)=∥F(u,v)∥ and an arguments matrix Φ(u,y).

Based on the shift Theorem of the unidimensional DFT, when two panoramic images have been captured from the same point on the floor, but having the robot different orientations around the vertical axis, both images present the same magnitudes matrix, and the arguments matrices can be used to estimate the relative orientation of the robot. Thanks to this property, the matrix A(u,y)=∥F(u,y)∥ can be considered as a visual descriptor of the robot position (as it is rotationally invariant), the matrix Φ(u,y) can be considered as a descriptor of the robot orientation (as it permits estimating this orientation), and the estimation of the position and the orientation can be addressed independently and sequentially.

To sum up, the position descriptor is the matrix $A\left(u,y\right)\in R^{k_{1}\times k_{2}}$ and the orientation descriptor is the matrix $\Phi \left(u,y\right)\in R^{k_{3}\times k_{4}}$. In the experiments, different sizes will be considered, to test separately the influence these parameters have on the accuracy and computational cost of the localization process.

### 2.2. Descriptors Based on Histograms of Oriented Gradients

The Histograms of Oriented Gradients (HOG) are local descriptors that have been used typically in computer vision and image processing to solve object detection tasks. HOG was initially described by Dalal and Triggs [45], who used it to detect persons in sequences of images. Afterwards, some researchers presented an improved version both in detection and computational cost [46]. Hofmeister et al. [47] made use of HOG to solve the localization of small mobile robots from low resolution images, in visually simple environments and when the orientation of the robot is similar to the orientation it had when the corresponding map image was captured. In [48], the same authors present a comparative of HOG with other appearance descriptors, applied to the localization of small robots in reduced environments, with similar results. Aslan et al. study the ability of HOG to handle occlusion in human tracking [49]. In addition, Neumann et al. use HOG, among other descriptors, for image-based vehicle detection and localization in an autonomous car [50].

Originally, HOG is built to describe local areas of a scene. We redefine it as a global appearance descriptor, using an exhaustive set of cells that covers the whole image and permits describing the global appearance. The version of HOG included in the comparative evaluation is presented in [51], where a global version of HOG is used to carry out map building and Monte Carlo localization in a large environment. When used to describe panoramic scenes, it presents rotational invariance and it also permits estimating the orientation of the robot.

In brief, from the initial panoramic image, a position and an orientation descriptor are obtained using the HOG philosophy. From the initial panoramic image $f\left(x,y\right)\in R^{N_{1}\times N_{2}}$ the magnitude and the orientation of the gradient are obtained and stored in the matrices M(x,y) and Θ(x,y), respectively. From now on, some sets of cells are defined upon the matrix Θ(x,y) to build the two descriptors. On the one hand, to build the position descriptor, a set of $k_{5}$ horizontal cells, whose width is equal to $N_{2}$ pixels, without overlapping, and covering the whole image are defined. For each cell, an orientation histogram with $b_{1}$ bins is compiled. During this process, each pixel in Θ(x,y) is weighted with the magnitude of the corresponding pixel in M(x,y). At the end of the process, the set of histograms are appended to compose the position descriptor ${\vec{h}}_{1}\in R^{k_{5}\cdot b_{1}\times 1}$. On the other hand, the orientation descriptor is built using the same steps, but considering a set of overlapped vertical cells, with a height equal to $N_{1}$ pixels, width equal to $l_{1}$ and distance between two consecutive cells equal to $d_{1}$. The number of vertical cells is $k_{6}=N_{2}/d_{1}$. After compiling a gradient orientation histogram for each cell, with $b_{2}$ bins and appending them, the result is the orientation descriptor ${\vec{h}}_{2}\in R^{k_{6}\cdot b_{2}\times 1}$.

The descriptor ${\vec{h}}_{1}$ is invariant against rotations of the robot in the ground plane so it can be considered as a visual descriptor of the robot position, and the information contained in ${\vec{h}}_{2}$ permits estimating the orientation of the robot with respect to a reference image.

### 2.3. Descriptors Based on Gist

The descriptors based on *gist* try to imitate the ability of the human perception system to recognize immediately a scene through the identification of specific regions stand out with respect to their neighborhood. This concept was introduced by Oliva and Torralba [52,53] with the idea of creating a low dimensional global image descriptor. More recent works make use of the concept of *prominence* together with *gist*. Siagian et al. [54] try to establish synergies between both concepts in a unique descriptor whose computational cost is relatively reduced. While these descriptors have been used thoroughly in classification tasks, the experience in mobile robotics localization is more sparse. Some related applications can be found in [55], where a localization and navigation system based on the *gist* and *prominence* concepts is presented; in [56], where *gist* descriptors, calculated over specific portions of a set of panoramic images, are used to solve a localization problem in urban areas; and in [57], where descriptors based on *gist* and dimensionally reduced by means of Principal Components Analysis are used to solve the loop closure problem in Simultaneous Localization and Mapping. In addition, Su et al. use *gist* in a localization framework to match keyframes, in combination with local descriptors to improve localization accuracy [58].

The description method we have included in this comparative analysis is based on the works of Siagian et al. [54] and is deeply described in [51]. It is built from orientation information, obtained by means of a bank of Gabor filters with different orientation, in some levels of resolution. First, two versions of the original panoramic image are considered: the original one and a new lower resolution version after applying a Gaussian low-pass filter and subsampling to a new size $0.5\cdot N_{1}\times 0.5\cdot N_{2}$. After that, both images are filtered with a bank of $m_{1}$ Gabor filters whose orientations are evenly distributed between 0 and 180 deg. Finally, to reduce the amount of information, the pixels in each resulting image are grouped into blocks, by calculating the average intensity of all the pixels contained in a block. The block division is chosen in an identical fashion than in the case of HOG. First, a set of $k_{7}$ horizontal blocks is defined to obtain the position descriptor ${\vec{g}}_{1}\in R^{2\cdot k_{7}\cdot m_{1}\times 1}$, which is invariant against rotations of the robot in the ground plane. Second, a set of $k_{8}$ vertical blocks with overlapping is defined to obtain the orientation descriptor ${\vec{g}}_{2}\in R^{2\cdot k_{8}\cdot m_{2}\times 1}$.

### 2.4. Descriptors Based on Wi-SURF

SURF [11] has been considered one of the most important local descriptors and it has been used in countless works as in [59] or [32] where it is used to solve localization indoors. The present study is focused on the performance of global appearance descriptors. For this reason, we propose an adaptation which is based on the work [60], which extracts a unique, global appearance descriptor per image, using the SURF philosophy. Throughout the paper, we will refer to this descriptor as Whole Image SURF (Wi-SURF).

Wi-SURF has been used in previous works for topometric localization [61] or for place recognition [40]. These works propose to obtain a unique vector $d\in R^{64}$ that contains gradient information of the entire image. Therefore, such a descriptor can be useful for place recognition, but does not contain enough information to estimate relative orientation. For this reason, we propose dividing the panoramic image into a set of evenly distributed square windows, with some overlapping between them. In each window, a SURF descriptor $d\in R^{64}$ is calculated and all the descriptors are concatenated, which leads to a global-appearance descriptor. This approach will enable us to solve not only the localization but also to estimate the relative orientation of the robot, as detailed in Section 3.4. The square windows are evenly distributed following the next parameters: $k_{9}$ is the number of horizontal cells in which the panoramic image is split and $sp_{1}$ the horizontal space between consecutive windows. The number of windows per cell will depend on the images’ width (512 columns in our experiments) so a total of $w_{1}=\frac{512}{sp_{1}}$ windows per cell are calculated. The width of the square window is equal to the height of the horizontal cell. After all, the size of the descriptor is $\vec{ws}\in R^{k_{9}\cdot w_{1}\cdot 64\times 1}$. This final descriptor will be used to estimate both position and orientation.

### 2.5. Descriptors Based on BRIEF-Gist

BRIEF-gist is a global appearance descriptor based on the local descriptor Binary Robust Independent Elementary Features (BRIEF). BRIEF was presented in [12] and used for different mobile robot applications [62,63]. Based on this local descriptor, a global appearance descriptor is presented in [64]. This approach is known as BRIEF-gist and it has been used for place recognition and loop closure detection in [40]. In the present work, we adapt this descriptor to be used with panoramic images in such a way that it permits calculating both relative distance and orientation in a localization task.

To implement the BRIEF-gist descriptor, the image is divided into $k_{10}\times w_{2}$ windows equally sized. Then, using the BRIEF description methodology, a set of ordered pairs of pixels is defined in each window, and the intensity of the second pixel of each pair is compared to the first one. If the difference is positive a 1 is added to the global descriptor, and a 0 if the difference is negative. As a result, a boolean vector is obtained. After this process, the resulting BRIEF-gist descriptor is $\vec{bg}\in R^{k_{10}\cdot w_{2}\times 1}$. This final descriptor is used to estimate both position and orientation.

### 2.6. Descriptors Based on Radon Transform

The Radon transform was proposed in [65]. Initially, it was used in different computer vision applications as a geometric shape descriptor, as in [66,67]. More recently, the Radon transform (RT) has been adapted to describe globally omnidirectional images and its performance was tested in [41], where descriptors based on the RT were used to solve the image retrieval problem, and in [23], where these descriptors were used to estimate relative altitude from images. The main advantage of this descriptor is that it can be calculated with raw omnidirectional images, as captured by the vision system (with no panoramic transformation).

Mathematically, the Radon transform consists of describing a function in terms of the projections of its linear integrals.

After applying the Radon transform, the image is transformed into a function $r_{im}\left(\Phi ,d\right)$, which is obtained after integrating the original function through several groups of parallel lines with distance to the origin *d* and different orientation Φ. The size of the new descriptor is $r_{im}\in R^{M_{x}\times M_{y}}$, $M_{x}$ is the number of orientations where Φ = {$\Phi_{1}$,$\Phi_{2}$,...,$\Phi_{M_{x}}$} and $M_{y}$ is the number of parallel lines.

**Figure 1:**
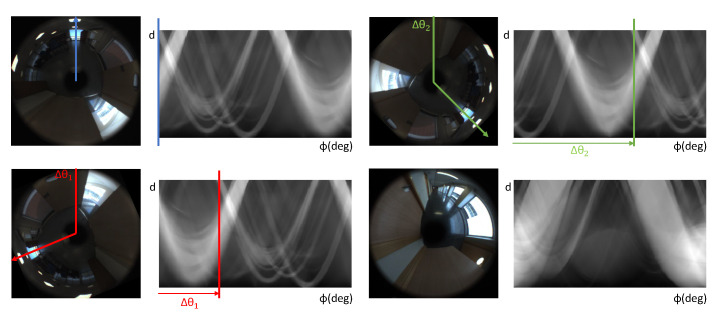
Shift property in the Radon transform.

When the Radon transform is applied to omnidirectional images, it is specially interesting its symmetry and the fact that the descriptor is horizontally shifted when the robot rotates [68], which allows us to obtain global appearance descriptors that can be used to estimate position and relative orientation. This property can be seen in Figure 1, where four omnidirectional images are shown; three of them have been taken from the same position but with different orientation and the other one has been taken from a different position. The figure clearly shows the effect of the orientation in the Radon transform and how different the result is if the image is from another room. If the robot rotates (Δθ) degrees, the new descriptor presents the same information as the original one, but it has been shifted *s* columns, *s* = (Δθ)· (M_x)/360. Thanks to this property, descriptors based on Radon transform contain position and orientation information of the robot.

To sum up, after applying the Radon transform to an omnidirectional image with size $N_{x}\times N_{x}$, a matrix $r\in R^{\frac{360}{p_{1}}\times 0.5\cdot N_{x}}$ is obtained. $p_{1}$ is the angle (deg.) between consecutive sets of lines along which the linear integrals are calculated. In the experiments, these matrices can be used in different ways in order to obtain proper uni-dimensional descriptors. Two different methods and different sizes will be considered to test the robustness of the descriptor in pose estimation. These methods and parameters are described in Section 3.

## 3. Solving the Absolute Localization Problem

In this work, we assume a visual model of the environment is previously available. To build this model, the robot has gone through the initially unknown environment (either in a tele-operated way or using any exploration algorithm [69,70]) and has captured a set of omnidirectional images from *n* points of view, defined by the poses ${\vec{p}}_{j}=\left(x_{j},y_{j},\theta_{j}\right),\;$j=1,⋯,n, to cover the whole environment to map. The model M is composed of the visual descriptors and the pose of the robot, stored for each capture position: $M=\{\left(D_{1},{\vec{p}}_{1}\right),\left(D_{2},{\vec{p}}_{2}\right),\cdots \left(D_{n},{\vec{p}}_{n}\right)\}$ where, in general, the description of each image consists of a position and an orientation descriptor $D_{j}=\left\{{\vec{d}}_{1j},{\vec{d}}_{2j}\right\}$ (in the case of *Wi-SURF* and *BRIEF-gist* the same vector is used as position and orientation descriptor, so $D_{j}=\left\{{\vec{d}}_{1j}\right\}$). The map building process using global appearance methods and omnidirectional imaging is thoroughly described in [39].

Once the model is built, the localization problem consists of estimating the pose of the robot. The problem is approached here as an absolute localization problem, i.e., no information on the previous position of the robot is considered, and only visual information is used. The robot captures a new image at time instant *t*, from an unknown pose ($f_{t}$, *test image*). Then, the descriptor of this image $D_{t}$ is computed and compared with the set of descriptors stored in the model. From this comparison, the position and orientation of the robot at time instant *t* are estimated. The next subsections detail these processes depending on the description method used.

### 3.1. Descriptors Based on the Discrete Fourier Transform

When a test image arrives, $A_{t}$ and $\Phi_{t}$ are calculated. Since the position descriptor is invariant against rotations of the robot in the ground plane, first, $A_{t}$ is used to estimate the position of the robot, by comparing it with the descriptors $A_{j},\;j=1,\cdots ,n$ and retaining the k-nearest neighbors. The position of the nearest neighbor $\left(x_{i},y_{i}\right)$ (*i* is the index of the nearest neighbor) can be considered as an estimation of the position of the robot at time instant *t*. Once the position of the robot has been estimated, the arguments matrix of the *test image*, $\Phi_{t}$, and the arguments matrix of the nearest neighbor, $\Phi_{i}$, are used to estimate the orientation of the robot, using the shift theorem of the DFT. The objective is to estimate the relative orientation $\theta_{ti}$ of the robot at time instant *t* with respect to the orientation the robot had when capturing the nearest neighbor, $\theta_{ti}=\theta_{t}-\theta_{i}$. The next steps are as follows:

1. A set of artificial rotations is applied to the *test image*. The shift theorem of the unidimensional DFT can be used to generate the argument matrices of the test image rotated siblings. The step between consecutive rotations is Δϕ. This is equivalent to making a shift of the columns of the panoramic image with a magnitude of *d* pixels, where $\Delta \phi =d\cdot 2\pi /N_{2}$. In the experiments, we consider $d=\{1,2,\cdots ,N_{2}-1\}$. This means that the angular step between consecutive artificial rotations is $\Delta \phi =2\pi /N_{2}$. This is the resolution of the method.
2. After this process, a set of $n_{rot}=2\pi /\Delta \phi$ arguments matrices are available at time instant *t*.
  (1) $${\left\{\Phi_{0},\Phi_{1},\cdots ,\Phi_{n_{rot}}\right\}}_{t}={\left\{\Phi_{\alpha }\right\}}_{t},\alpha =0,\ldots ,n_{rot}$$
3. The Hadamard product of the matrix $\Phi_{t}$ and every matrix $\Phi_{\alpha }$ is calculated. The sum of the components of each resulting matrix is obtained, and the result is an array of data:
  (2) $${\left\{m_{0},m_{1},\cdots ,m_{n_{rot}}\right\}}_{t}={\left\{m_{\alpha }\right\}}_{t},\alpha =0,\ldots ,n_{rot}$$
4. The estimated relative rotation is the α value whose coefficient $m_{\alpha }$ presents the maximum value.
  (3) $$\alpha ={arg\;max}_{\alpha }\left\{m_{\alpha }\right\}$$
  (4) $$\theta_{ti}=\frac{2\pi \alpha }{n_{rot}}$$
  where $\theta_{ti}$ is the relative orientation between the image $im_{t}$ and the nearest neighbor of the map, $im_{i}$. This way, the absolute orientation of the robot at time instant *t* can be calculated as:
  (5) $$\theta_{t}=\theta_{i}+\theta_{ti}$$
  In this equation, $\theta_{i}$ is the orientation that the robot had when the map image $im_{i}$ was captured, with respect to the global reference system.

In the experiments, the parameters of the Fourier Signature to optimize are the size of the module matrix ($k_{1}$ and $k_{2}$) and the size of the arguments matrix ($k_{3}$ and $k_{4}$) to reach a balance between the accuracy in the estimation of the position and orientation and the computational cost of the algorithms.

### 3.2. Descriptors Based on Histograms of Oriented Gradients

Once the test image $im_{t}$ has been captured, the descriptors ${\vec{h}}_{1t}$ and ${\vec{h}}_{2t}$ are calculated. First, the k-nearest neighbors to ${\vec{h}}_{1t}$ among the set of descriptors ${\vec{h}}_{1j},\;j=1,\cdots ,n$ are calculated and extracted. The position $\left(x_{i},y_{i}\right)$ of the nearest neighbor *i* is an estimation of the position of the robot at time instant *t*.

Later, the orientation is calculated by comparing the vector ${\vec{h}}_{2t}$ with the vector ${\vec{h}}_{2i}$. With this aim, a set of artificial rotations is calculated using the vector ${\vec{h}}_{2t}$ and later, the scalar product between the resulting vector after each rotation and the vector ${\vec{h}}_{2i}$ is calculated. To simulate a rotation of the vector ${\vec{h}}_{2t}$, the circular shift must be a multiple of $b_{2}$ positions ($b_{2}$ is the number of bins per histogram). A shift of $b_{2}$ positions equals a rotation of the robot $\Delta \phi =2\pi d_{1}/N_{2}$ radians (this is the angular resolution of the method), where $d_{1}$ is the distance between two consecutive vertical cells.

Finally, the estimated relative orientation $\theta_{ti}$ of the robot is the angle that corresponds to the rotated version of the vector ${\vec{h}}_{2t}$ which presents a higher scalar product with ${\vec{h}}_{2i}$.

### 3.3. Descriptors Based on Gist

The processes to estimate the position and orientation are identical to those presented in the case of HOG. Once captured the test image $im_{t}$, the descriptors ${\vec{g}}_{1t}$ and ${\vec{g}}_{2t}$ are calculated. First, ${\vec{g}}_{1t}$ is compared to ${\vec{g}}_{1j},\;j=1,\cdots ,n$ and the k-nearest neighbors are calculated. From them, the position ${\left(x,y\right)}_{i}$ of the nearest neighbor *i* is considered an estimation of the position of the robot at time instant *t*. After that, the orientation is calculated by comparing the vector ${\vec{g}}_{2t}$ with the vector ${\vec{g}}_{2i}$. With this aim, successive artificial rotations are calculated, using the vector ${\vec{g}}_{2t}$ and later, the scalar product between each rotated version and the vector ${\vec{g}}_{2i}$ is obtained. To make an artificial rotation of the vector ${\vec{h}}_{2t}$, the magnitude of the circular shift must be a multiple of $m_{2}$ ($m_{2}$ is the number of components of each vertical block). Every shift equals a rotation of $\Delta \phi =2\pi d_{2}/N_{2}$ radians (this is the angular resolution of the method), where $d_{2}$ is the distance between two consecutive vertical blocks in the descriptor.

The resulting orientation $\theta_{ti}$ is the angle that corresponds to the rotated version of ${\vec{g}}_{2t}$ that presents the highest scalar product with ${\vec{g}}_{2i}$.

### 3.4. Descriptors Based on Wi-SURF

Once the test image $im_{t}$ is taken, the descriptor ${\vec{ws}}_{t}$ is obtained. First, this descriptor is compared with the descriptors ${\vec{ws}}_{j},j=1,\cdots ,n$, to calculate the relative orientation between the test descriptor and the descriptors in the model. To estimate the relative orientation, some artificial rotations are added to ${\vec{ws}}_{t}$ and the distance between the resulting descriptor after each rotation and ${\vec{ws}}_{j}$ is calculated. To simulate an artificial rotation of ${\vec{ws}}_{t}$, a circular shift is applied, which must be a multiple of 64 positions (the SURF descriptor of each window contains 64 components) and $w_{1}$ (number of windows). The 64-position shift of the descriptor equals to a rotation of the robot $\Delta \phi =2\cdot \pi \cdot sp_{1}/N_{2}$ radians (and therefore, this is the angular resolution of the method). Once the relative orientation between the test descriptor and each of the descriptors in the model has been calculated, each descriptor ${\vec{ws}}_{j}$ is shifted in such a way that the resulting descriptor has the same orientation as ${\vec{ws}}_{t}$.

Once all the descriptors are supposed to be in the same orientation, the k-nearest neighbors to ${\vec{ws}}_{t}$ are calculated among the set of descriptors in the model (once they are equally oriented with respect to ${\vec{ws}}_{t}$). The position ($x_{i},y_{i}$) of the nearest neighbor *i* is an estimation of the position of the robot at time *t*. The orientation between them has been calculated previously and the corresponding angle $\theta_{ti}$ is the relative orientation estimated between the test vector and the vector evaluated from ${\vec{ws}}_{j}$.

### 3.5. Descriptors Based on BRIEF-Gist

Firstly, the relative orientation between images is estimated. The descriptor ${\vec{bg}}_{t}$ is calculated from the test image $im_{t}$, and the relative orientation between it and each of the descriptors ${\vec{bg}}_{j},j=1,\cdots ,n$ is estimated. To estimate it, successive artificial rotations are applied to ${\vec{bg}}_{t}$, the scalar product between the resulting vector after each rotation and ${\vec{bg}}_{j}$ is calculated and the minimum is retained. To simulate an artificial rotation of ${\vec{bg}}_{t}$, the circular shift must be a multiple of $w_{2}$ (number of windows in each cell). As explained in Section 2.5, to calculate this descriptor the image is divided into $k_{12}\times w_{2}$ windows, so the angular resolution of the method is determined by the number of windows $w_{2}$. Every $w_{2}$ shift is equal to a rotation of the robot $\Delta \phi =2\cdot \pi /w_{2}$ radians.

After estimating the relative orientation, each descriptor in the model ${\vec{bg}}_{j}$ is rotated such that the resulting descriptor has the same orientation than ${\vec{bg}}_{t}$. Then the k-nearest neighbors to ${\vec{bg}}_{t}$ are calculated among the set of rotated descriptors in the model. The position ($x_{i},y_{i}$) of the nearest neighbor *i* is an estimation of the position of the robot at time *t*. The relative orientation between them has been calculated previously and the corresponding angle $\theta_{ti}$ is the difference of orientation estimated between the test vector and the vector evaluated from ${\vec{ws}}_{j}$.

### 3.6. Descriptors Based on the Radon Transform

In the present work, we process this descriptor using two different methods to retrieve both position and orientation.

#### 3.6.1. Radon–Fourier Method

After applying the Radon transform, a matrix $r\in R^{\frac{360}{p_{1}}\times 0.5\cdot N_{x}}$ is obtained. Then, the Fourier Signature of this matrix is calculated. As a result of this second transformation, a matrix of magnitudes ${A_{\mathrm{RT}}}_{j}\in R^{\frac{360}{p_{1}}\times k_{11}}$ and a matrix of arguments ${\Phi_{RT}}_{j}\in R^{\frac{360}{p_{1}}\times k_{12}}$ are obtained. As in the case of the descriptors based on the DFT, ${A_{\mathrm{RT}}}_{j}$ is used as position descriptor and ${\Phi_{RT}}_{j}$ is used as an orientation descriptor. $k_{11}$ is the number of columns taken for the position descriptor ${A_{\mathrm{RT}}}_{j}$ and $k_{12}$ is the number of columns taken for the orientation descriptor ${\Phi_{RT}}_{j}$. To estimate the position and orientation, we use the same process as in the descriptors based on the discrete Fourier transform, presented in the Section 3.1.

#### 3.6.2. Radon–POC Method

This method uses directly the matrix obtained after applying the Radon transform ( $r\in R^{\frac{360}{p_{1}}\times 0.5\cdot N_{x}}$) as the image descriptor ${r_{poc}}_{j}$. To compare two descriptors, Phase Only Correlation (POC) is used. This operation outputs a correlation coefficient that allows us to estimate the similarity between two matrices and their relative shift.

To sum up, Table 1 shows the parameters whose influence will be studied in the comparative evaluation. After that, Table 2 gives details of the contents of the model when we consider each description method.

## 4. Experimental Setup

This section describes the experimental setup. First, the sets of images used to carry out the experiments are presented. Second, a variety of phenomena (noise and occlusions) to test the robustness of the algorithms are described.

### 4.1. Sets of Images

All the experiments are carried out with two sets of images captured by ourselves [71]. A catadioptric vision system is used to capture the images. It is composed of an *Imaging Source DFK 21BF04* camera pointing towards an *Eizoh Wide 70* hyperbolic mirror, with their axes aligned. This system captures omnidirectional images which are preprocessed to obtain cylindrical projections (panoramic images) with size $N_{1}\times N_{2}=128\times 512$ pixels.

The first set of images is named the *training set* and it is composed of 872 panoramic images captured on a dense grid of points of 40×40 cm, covering the whole floor of a building of Miguel Hernández University (Spain), including 6 different rooms. The *training set* will be used to build a visual model of the environment. Different grid sizes will be considered along the experiments.

The second set is named the *test set* and it contains 1232 images captured in all the rooms, with different orientations. To capture these images, 77 positions were defined on some half-way points among the grid positions, and 16 images per position were captured, with different robot orientations, to cover the whole circumference. These images were captured in different times of day and with changes in the position of some objects, doors, etc., to reflect the natural variability of the visual information in real working environments. The *test set* will be used during the process of localization and orientation estimation, to test the goodness of each description method and the influence of the main parameters. This environment is very prone to *perceptual aliasing*, which means that two images captured from two positions which are far away may have a similar visual appearance. Global appearance descriptors must cope with this phenomenon as it frequently happens in indoor environments.

**Figure 2:**
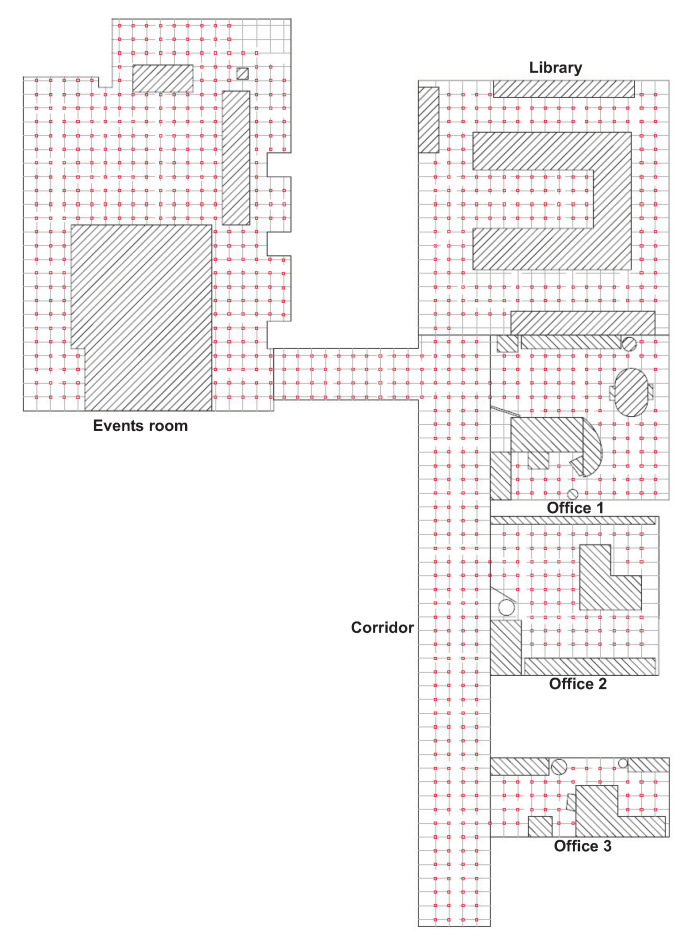
Bird’s eye view of the capture points of the training set of images. The size of the grid is 40×40 cm.

**Figure 3:**
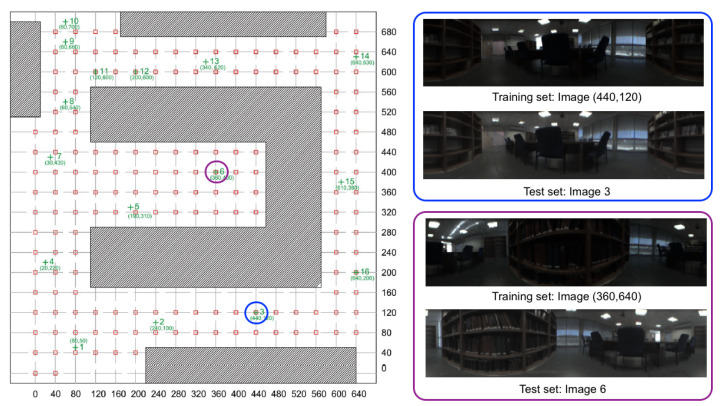
Library. Bird’s eye view of the capture points of the training set of images. The size of the grid is 40×40 cm.

**Figure 4:**
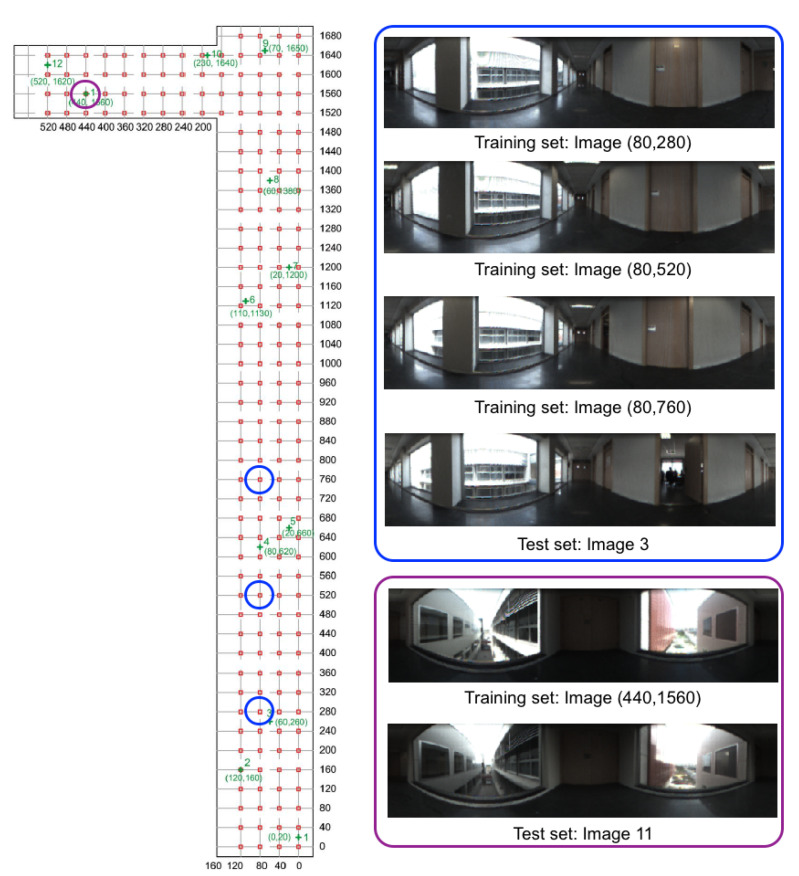
Corridor. Bird’s eye view of the capture points of the training set of images. The size of the grid is 40×40 cm.

Figure 2 shows a bird’s eye view of the environment and the capture points of the training images. As an example, Figure 3 shows the library, the capture points of the training (red) and test (green) images and some sample training and test images captured in close points. The effect of changes in lighting conditions and changes of orientation can be appreciated. Other sample space is shown in Figure 4 (corridor). The effect of *visual aliasing* is clearly shown. In addition, the test image 3 shows an example of changes in the environment (open door with respect to the training images).

### 4.2. Addition of Noise and Occlusions

The test images reflect some of the most habitual undesired effects in real working environment: changes in lighting conditions, in the position and state of some objects and perceptual aliasing. Additionally, two other phenomena are considered in the experiments: noise and occlusions.

**Figure 5:**
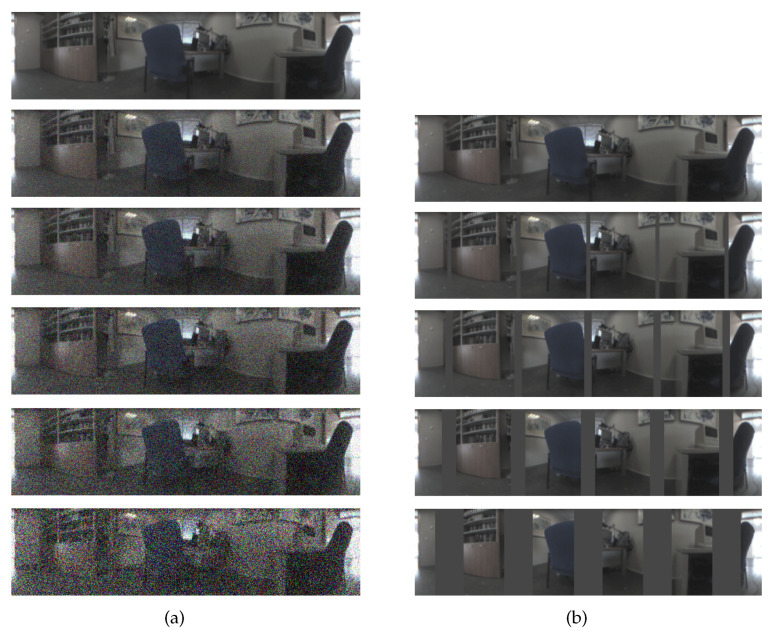
Sample image from the training test with (**a**) different levels of added Gaussian noise ($\sigma^{2}=\left\{0,0.0025,0.05,0.01,0.02,0.05\right\}$) and (**b**) sequence of occlusions considered ({0,5,10,20,40}%).

First, to test the influence of noise due to the nature of the acquisition system, noise with Gaussian distribution is considered, with null average value and several variance values, to consider different noise levels: $\sigma^{2}=\left\{0,0.0025,0.05,0.01,0.02,0.05\right\}$. Along the rest of the paper, these levels of noise are named noises 0, 1, 2, 3, 4 and 5, respectively. Figure 5a shows a test image with these levels of added noise. In the most extreme case, the visual appearance of the image is seriously altered.

Second, the presence of persons or other robots in the environment may occlude partially and temporarily the visual information. Working with panoramic images constitutes an advantage as far as occlusions are concerned. However, they may hide some relevant features with respect to the visual information stored in the map and put in risk the localization process. To model this effect, several levels of occlusion have been added artificially to the images, considering several vertical bars that produce different levels of occlusion, considering {0,5,10,20,40}% of the whole image occluded. Along the rest of the paper, these levels are named occlusions 0, 1, 2, 3, 4 and 5, respectively. Figure 5b shows a test image with these levels of added occlusion. In the most extreme case, 40% of the visual information is lost.

## 5. Results and Discussion

In this section, an exhaustive bank of experiments is proposed to test the performance of the global appearance descriptors included in the comparative evaluation and the influence of the main parameters in the accuracy and computational cost of the localization process. The experiments have been structured in four subsections. First, in Section 5.1, the ability of each descriptor to find the nearest neighbor of the model, in ideal conditions (considering neither noise nor occlusions) is tested. After that, the problem of position estimation is solved, including also the study of performance with these effects (Section 5.2). Third, in Section 5.3, the problem of orientation estimation is considered. Finally, Section 5.4 studies the relative performance of the descriptors with a trajectory-like dataset.

### 5.1. Image Retrieval Problem

During the localization process, the first step consists of comparing the localization descriptor of the test image with all the localization descriptors in the map and obtaining the k-nearest neighbors. Taking this fact into account, in this section we evaluate the ability of each description method to calculate correctly the first nearest neighbor (i.e., to identify correctly the position of the model which is geometrically the nearest one to the test position). It is known as the *image retrieval problem*.

To obtain the k-nearest neighbors of a test image descriptor, several kinds of distances can be considered. In this study, four distance measurements are implemented and compared. In the next lines, these distances are formalized. Considering $\vec{r}=\left\{r_{i}\right\}$, i=1,⋯,l and $\vec{s}=\left\{s_{i}\right\},i=1,\cdots ,l$, the two data vectors whose distance we want to obtain:

1. Weighted metric distance:
  (6) $$dist_{p}\left(\vec{r},\vec{s}\right)={\left(\sum_{i=1}^{l}\omega_{i}\cdot {\left|r_{i}-s_{i}\right|}^{p}\right)}^{\frac{1}{p}}$$
  If we consider $\omega_{i}=1$, i=1,⋯,l, the Minkowski distance is obtained. Two particular cases will be considered: $dist_{1}$ (*Manhattan* distance), which is defined from the Minkowski distance with p=1, and $dist_{2}$ (Euclidean distance), doing p=2.
2. Pearson correlation coefficient. It is a similitude coefficient that can be obtained as:
  (7) $$sim_{Pea}\left(\vec{r},\vec{s}\right)=\frac{{\vec{r}}_{d}^{T}\cdot {\vec{s}}_{d}}{|{\vec{r}}_{d}\left|\right|{\vec{s}}_{d}|}$$
  where ${\vec{r}}_{d}=\left[r_{1}-\bar{r},\cdots ,r_{l}-\bar{r}\right]$ and ${\vec{s}}_{d}=\left[s_{1}-\bar{s},\cdots ,s_{l}-\bar{s}\right]$, $\bar{r}=\frac{1}{l}\sum_{j}r_{j}$, $\bar{s}=\frac{1}{l}\sum_{j}s_{j}$. It takes values in the range [−1,+1]. From this similitude coefficient, a distance measure can be defined as:
  (8) $$dist_{3}\left(\vec{r},\vec{s}\right)=1-sim_{Pea}\left(\vec{r},\vec{s}\right)$$
3. Inner product: It is also a similitude coefficient that can be calculated as the scalar product between the two vectors to compare.
  (9) $$sim_{cos}\left(\vec{r},\vec{s}\right)=\frac{{\vec{r}}^{T}\cdot \vec{s}}{|\vec{r}\left|\right|\vec{s}|}$$
  As shown in the equation, $\vec{r}$ and $\vec{s}$ are usually normalized. In this case, this measure is known as *cosine similitude* and takes values in the range [−1,+1]. The corresponding distance value is:
  (10) $$dist_{4}\left(\vec{r},\vec{s}\right)=1-sim_{in}\left(\vec{r},\vec{s}\right)$$

Therefore, the four distance measurements compared along this section are: $dist_{1}$ (Manhattan distance), $dist_{2}$ (Euclidean distance), $dist_{3}$ (Pearson correlation-based distance) and $dist_{4}$ (cosine similitude-based distance).

**Figure 6:**
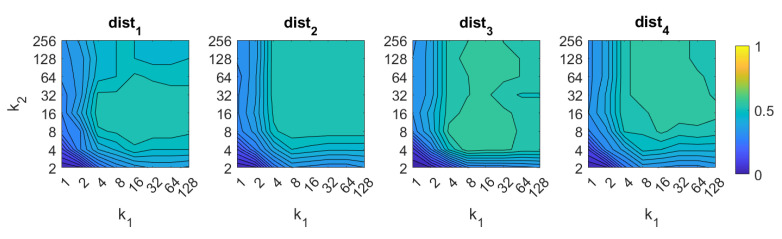
FS image retrieval problem. Success rate of the method. $k_{1}$ and $k_{2}$ are, respectively, the number of rows and columns of the descriptor (Table 1).

**Figure 7:**
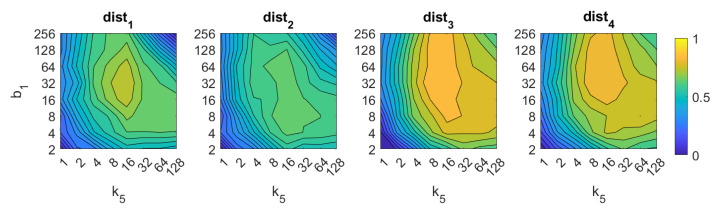
HOG image retrieval problem. Success rate of the method. $k_{5}$ is the number of horizontal cells and $b_{1}$ the number of bins per histogram (Table 1).

**Figure 8:**
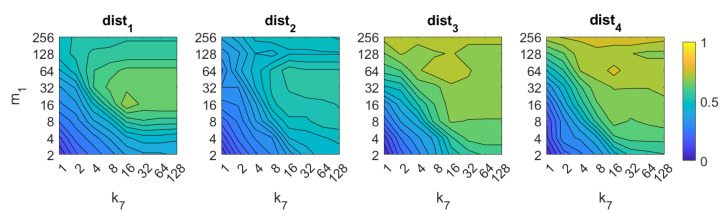
*Gist* image retrieval problem. Success rate of the method. $k_{7}$ is the number of horizontal blocks and $m_{1}$ the number of Gabor filters to build the descriptor (Table 1).

**Figure 9:**
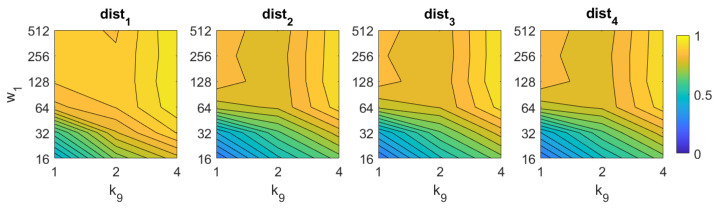
WS image retrieval problem. Success rate of the method. $k_{9}$ is the number of horizontal cells and $w_{1}$ the number of windows per cell (Table 1).

**Figure 10:**
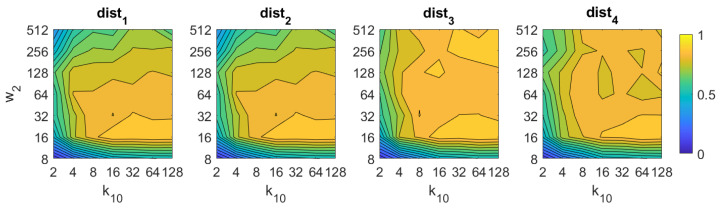
BG image retrieval problem. Success rate of the method. $k_{10}$ is the number of horizontal cells and $w_{2}$ the number of windows per cell (Table 1).

**Figure 11:**
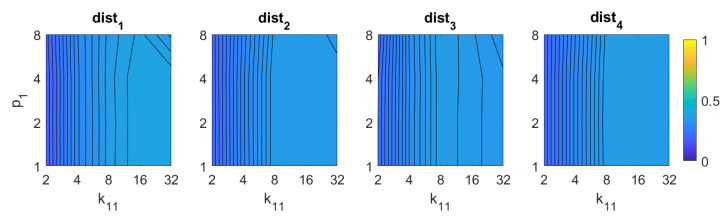
RT–F image retrieval problem. Success rate of the method. $k_{11}$ is the number of blocks and $p_{1}$ the relative angle (deg) between the lines in each set (Table 1).

**Figure 12:**
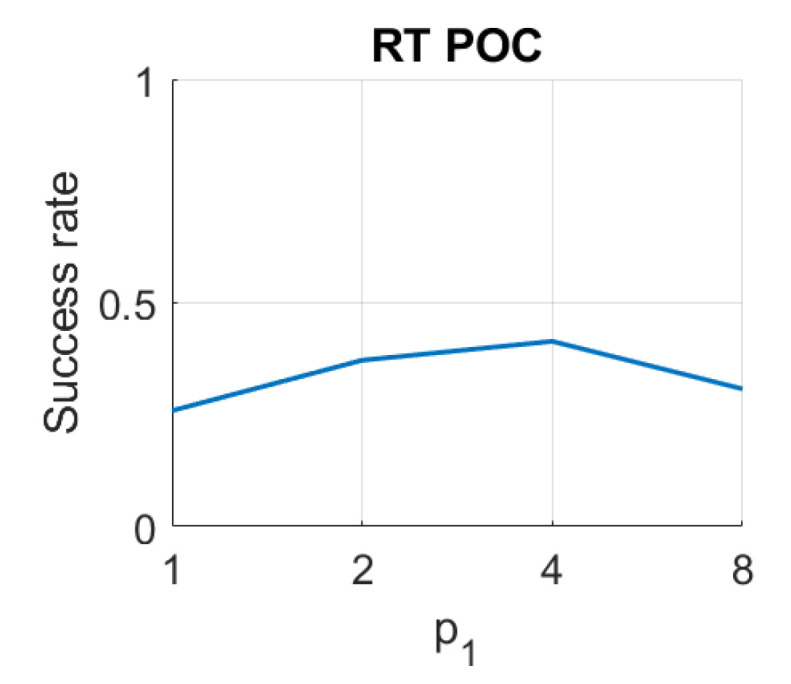
RT–POC image retrieval problem. Success rate of the method. $p_{1}$ is the relative angle (deg) between the lines en each set (Table 1).

First, the success rate of each algorithm is studied. It assesses the ability of the localization algorithm to calculate correctly the first nearest neighbor (i.e., to identify correctly the position of the model which geometrically the nearest one to the test position). Figure 6, Figure 7, Figure 8, Figure 9, Figure 10, Figure 11 and Figure 12 show the success rate, expressed on a per unit base. For comparative purposes, all the results are expressed in the same color scale.

The behavior of the FS changes slightly depending on the distance measurement used. The best results are obtained with $dist_{3}$ and $dist_{4}$ with an intermediate number of rows and an intermediate to high number of columns. In all cases, an excessively low number of rows and/or columns provides bad results. The best accuracy is 60%, and it is obtained with the distance $dist_{3}$ and $k_{1}=k_{2}=8$.

About HOG, the best results are also obtained with distances $dist_{3}$ and $dist_{4}$. In both cases, the number of horizontal cells $k_{5}$ must be an intermediate value, around 16. A higher number does not improve the accuracy of the method. The number of bins per histogram $b_{1}$ must take values from intermediate to high, starting from 16. In the case of distances $dist_{1}$ and $dist_{2}$, an excessively high number of cells and bins also provides remarkably bad results. The best accuracy is 89%, and it is obtained with the distance $dist_{3}$ and $k_{5}=8,b_{1}=32$.

In the case of *gist*, the best results are obtained again using the distances $dist_{3}$ and $dist_{4}$. In these cases, the accuracy increases as the number of masks $m_{1}$ does. It is not necessary a high number of masks $m_{1}$ to obtain good results. The best accuracy is 89%, and it is obtained with the distance $dist_{3}$ and $k_{7}=32,m_{1}=256$.

In the case of *Wi-SURF*, the best results are obtained using the distances $dist_{1}$ and $dist_{3}$. In these cases, the image retrieval problem is solved with a better rate when using high values of $k_{9}$ (around 4). The process performs correctly with intermediate and high number of windows per cell $w_{1}$, starting from 128. The best rate is 97%, and it is obtained with the distance $dist_{1}$ and $k_{9}=4,w_{1}=512$.

If we analyze now *BRIEF-gist*, the best results are obtained using the distances $dist_{3}$ and $dist_{4}$. A high number of horizontal cells $k_{10}$ is needed to obtain suitable results, about 64. A high number of windows $w_{2}$ does not improve the results necessarily, but remarkably bad results are obtained using low values of $k_{10}$ or $w_{2}$. The best accuracy obtained with *BG* is 93%, and it is obtained with the distance $dist_{3}$ and $k_{10}=64,w_{2}=16$.

Finally, in the case of *RT*, the results are not competitive if they are compared with the rest of the descriptors. On the one hand, using the Radon transform along with the Fourier Signature, the best results are obtained with the distances $dist_{1}$ and $dist_{4}$. In this case the parameters have less relevance on the results, but in general, high values of $k_{11}$ and low values of $p_{1}$ lead to better rates. Using *RT–F*, the best accuracy is 39%, and it is obtained with the distance $dist_{1}$ and $k_{11}=32,p_{1}=1$. On the other hand, using the POC method, the best rate is 41% obtained with $p_{1}=4$.

Analyzing globally these figures, Wi-SURF is the description algorithm that presents the best absolute success rate, when it is used along with $dist_{1}$. In general, the distance $dist_{3}$ performs much better than the rest in almost all the cases. HOG, *gist* and *BRIEF-gist* are also acceptable methods. Taking into account the challenging characteristics of the environment, they provide remarkably good results.

**Figure 13:**
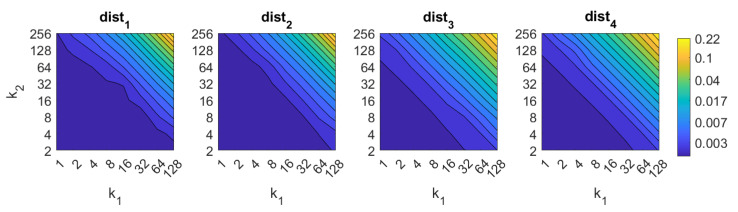
FS image retrieval problem. Computational time.

**Figure 14:**
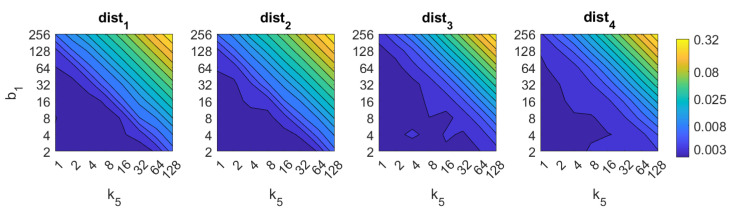
HOG image retrieval problem. Computational time.

**Figure 15:**
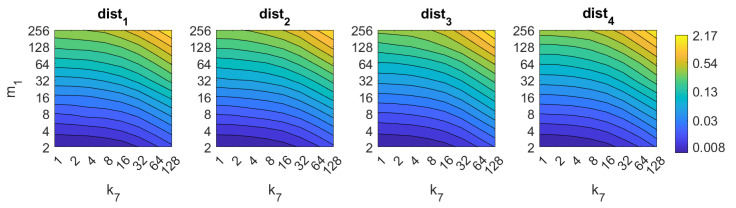
*Gist* image retrieval problem. Computational time.

**Figure 16:**
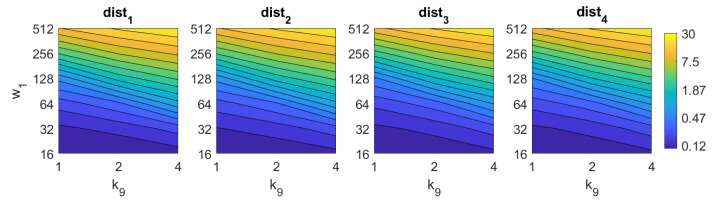
WS image retrieval problem. Computational time.

**Figure 17:**
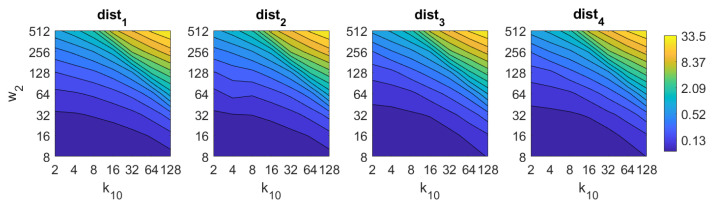
BF image retrieval problem. Computational time.

**Figure 18:**
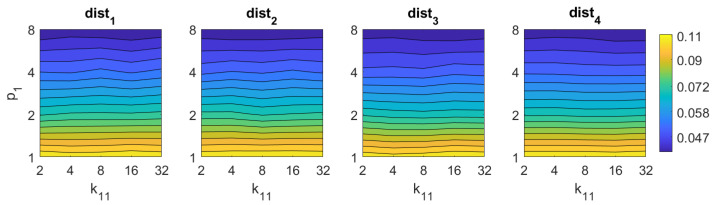
RT–F image retrieval problem. Computational time.

**Figure 19:**
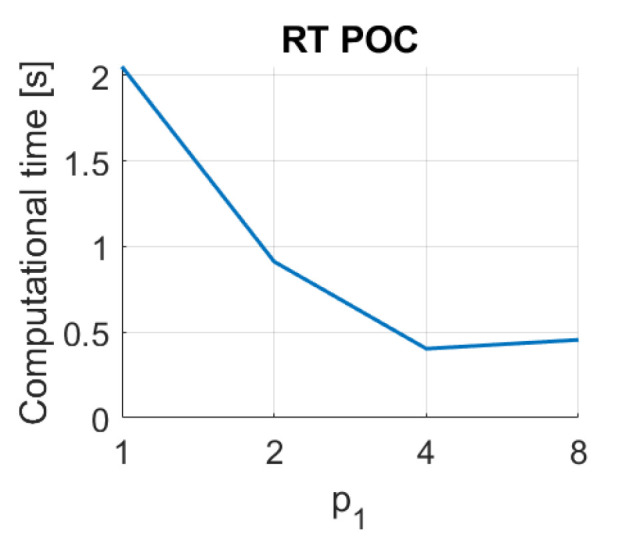
RT–POC image retrieval problem. Computational time.

Apart from the success rate, it is also worth studying the computational cost of the process, to evaluate whether the localization task could be carried out in real time. Figure 13, Figure 14, Figure 15, Figure 16, Figure 17, Figure 18 and Figure 19 show the necessary time to obtain the nearest neighbor, depending on the size of the position descriptor. The average value after all the experiments is shown, expressed in seconds. A logarithmic scale has been used to represent efficiently the time in the color scale.

The experiments have been carried out with a CPU Intel Core i7-9700^®^ at 3 GHz and using the mathematical tool Matlab^®^. These time results are not absolute, they depend of the computer which runs the process. They are comparable because all the calculations have been done with the same machine.

First, FS is the quicker algorithm. The average time per *test image* is under 0.02 s for the majority of configurations. Only when both $k_{1}$ and $k_{2}$ take high values, the computational time takes values around 0.22 s. Both parameters have a similar influence on the computational cost. Second, the computational cost of HOG is slightly higher than FS, depending on the configuration of the parameters. Both parameters $b_{1}$ and $k_{3}$ have similar influence on this time. When their values are high it is possible to find some results where the runtime takes around 0.32 s. Third, *gist* is computationally more an expensive algorithm. $m_{1}$ has a strong influence on the necessary time. A high number of masks along with high values of $k_{7}$ make the time per image to take values around 2.1 s. Anyway, it is possible to find configurations that provide acceptable computational times with a lower number of components.

The second group of descriptors, in which each descriptor should be shifted until finding the relative orientation before retrieving the image, are considerably slower. On the one hand, *Wi-SURF* needs more than 2 s with most of the configurations. $w_{1}$ has more influence on the computational time so, as far as possible, it is better to avoid high values of this parameter. High values of the parameters can lead to times up to 30 s. On the other hand, *Wi-SURF* is the computationally most expensive method. $w_{2}$ has a strong influence on the process, and produces times about 33.5 s.

Finally, the method based on the Radon transform and Fourier performs quickly, with times typically under 0.1 s. The method based on Radon transform and POC leads to times around 0.5 s with some configurations of $p_{1}$. Notwithstanding that, since the descriptors based on the Radon transform have proved to perform poorly in the image retrieval task, these descriptors are not included in subsequent analyses.

### 5.2. Estimation of the Position

The second set of experiments assesses the ability of each description method to estimate correctly the position of the robot, when noise or occlusions are present, depending on the size of the descriptor and the type of distance considered.

For each test image, the position descriptor is obtained and compared with all the position descriptors in the map. The 1st nearest neighbor is then retained, using any distance measurement. In the cases that it is possible, a k-d tree has been implemented to make efficiently this search. After obtaining the nearest neighbor, the Euclidean distance between the real position of the robot at time instant *t* and the position of the nearest neighbor is considered the position error.

**Figure 20:**
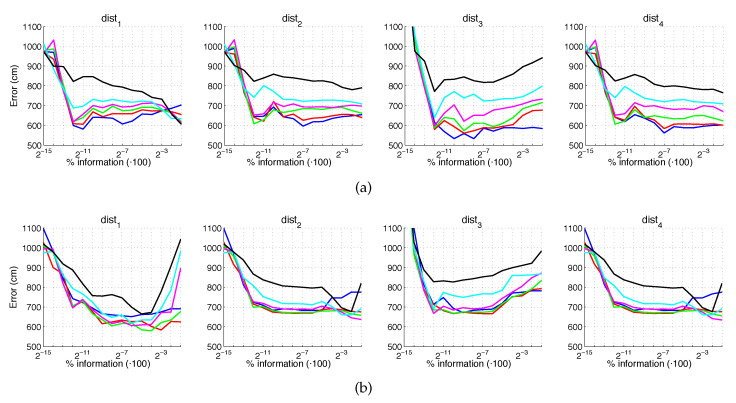
FS average localization error with noise: (**a**) no filter and (**b**) homomorphic filtering. Legend: — Original, — Noise 1, — Noise 2, — Noise 3, — Noise 4, — Noise 5.

**Figure 21:**
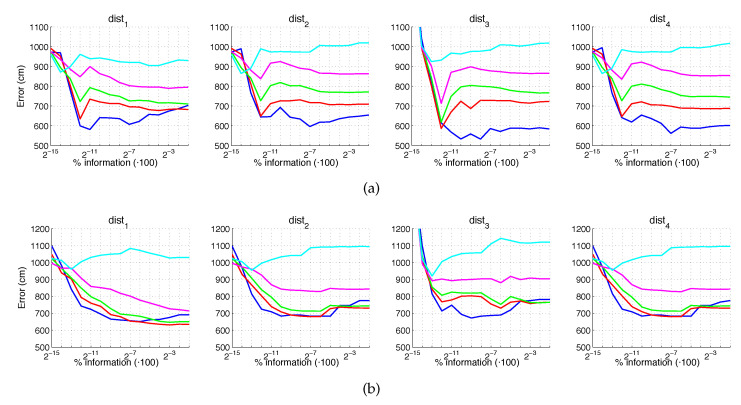
FS average localization error with occlusions: (**a**) no filter and (**b**) homomorphic filtering. Legend: — Original, — Occlusion 1, — Occlusion 2, — Occlusion 3, — Occlusion 4.

Figure 20 and Figure 21 present the results obtained with the Fourier Signature considering the presence of noise or occlusions, respectively, in the test images. In these figures, first, no filter is considered (a) and second, a homomorphic filtering is carried out both to the reference and the test images (b). The result is expressed then as the average position error, expressed in cm after considering the 1232 test images. The horizontal axis expresses the percentage of information considered per configuration, expressed in logarithmic scale. The ticks of each graphical representation are $\left\{2^{-15},2^{-14},2^{-13},\cdots ,2^{-2},2^{-1}\right\}$ which correspond, respectively, to the next percentages of information {0.003%,0.06%,0.12%,⋯,25%,50%}. These percentages express the information contained in each descriptor with respect to the information contained in each original panoramic image $\left(\frac{k_{1}\cdot k_{2}}{N_{1}\cdot N_{2}}\cdot 100\right)$. In general, the use of homomorphic filtering worsens the results. As expected, the higher the level of noise, the higher the error. However, $dist_{1}$ and $dist_{2}$ present a more robust behavior when noise is present. About the presence of occlusions, the FS descriptor is quite sensitive to this phenomenon and the results worsen substantially when the percentage of occlusion increases.

**Figure 22:**
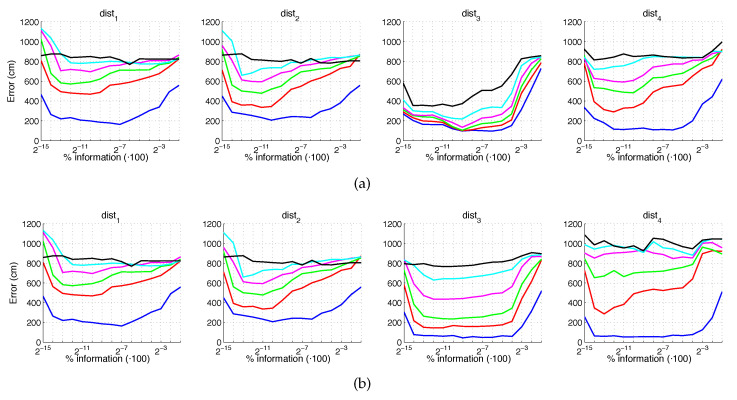
HOG average localization error with noise: (**a**) no filter and (**b**) homomorphic filter. Legend: — Original, — Noise 1, — Noise 2, — Noise 3, — Noise 4, — Noise 5.

**Figure 23:**
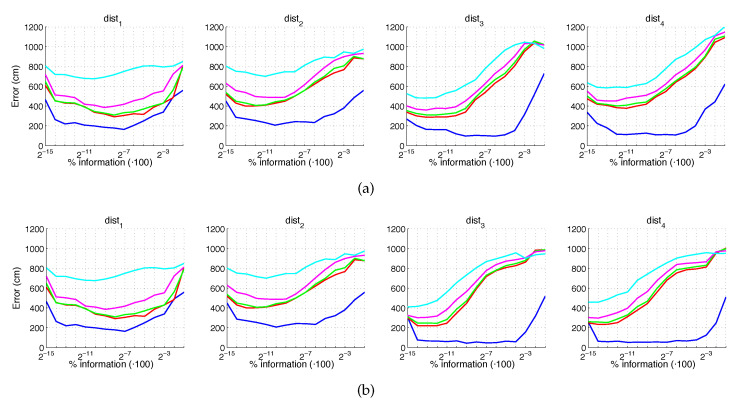
HOG average localization error with occlusions: (**a**) no filter and (**b**) homomorphic filter. Legend: — Original, — Occlusion 1, — Occlusion 2, — Occlusion 3, — Occlusion 4.

Figure 22 and Figure 23 present the results obtained with the Histogram of Oriented Gradients considering the presence of noise or occlusions, respectively, in the test images. In these figures, first, no filter is considered (a) and second, a homomorphic filtering is carried out both to the reference and the test images (b). Like in the case of FS, the percentages in the horizontal axis express the information contained in each descriptor with respect to the information contained in each panoramic image. In the case of HOG they can be obtained as $\left(\frac{k_{5}\cdot b_{1}}{N_{1}\cdot N_{2}}\cdot 100\right)$. In presence of noise, the use of homomorphic filtering only improves the results with distances $dist_{3}$ and $dist_{4}$ and with low level of noise. Intermediate percentages of information tend to present the best absolute results so it is not necessary to store a big quantity of information during the construction of the descriptor. In presence of noise, the best absolute results are obtained with $dist_{3}$, no filter and intermediate quantity of information. Comparing to the other description methods, HOG stands out thank to its robustness against presence of occlusions in the test images.

**Figure 24:**
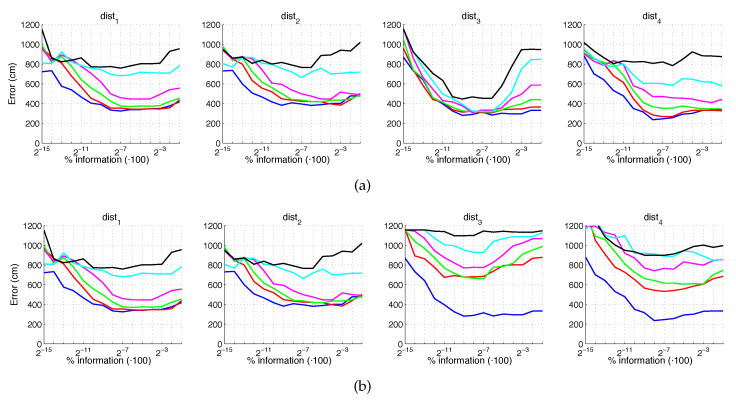
*Gist* average localization error with noise: (**a**) no filter and (**b**) homomorphic filtering. Legend: — Original, — Noise 1, — Noise 2, — Noise 3, — Noise 4, — Noise 5.

**Figure 25:**
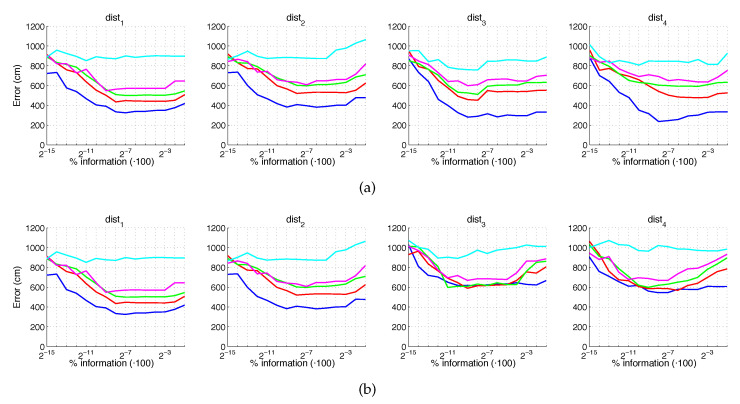
*Gist* average localization error with occlusions: (**a**) no filter and (**b**) homomorphic filtering. Legend: — Original, — Occlusion 1, — Occlusion 2, — Occlusion 3, — Occlusion 4.

Additionally, Figure 24 and Figure 25 present the results obtained with *gist* considering the presence of noise or occlusions, respectively, in the test images. In these figures, first, no filter is considered (a) and second, a homomorphic filtering is carried out both to the reference and the test images (b). Like in the case of FS, the percentages of information contained in each descriptor with respect to the information contained in each panoramic image can be obtained as $\left(\frac{2\cdot k_{7}\cdot m_{1}}{N_{1}\cdot N_{2}}\cdot 100\right)$. The use of homomorphic filtering does not improve the localization results in any case. In the presence of noise, $dist_{3}$ presents the best results when considering an intermediate percentage of information.

**Figure 26:**
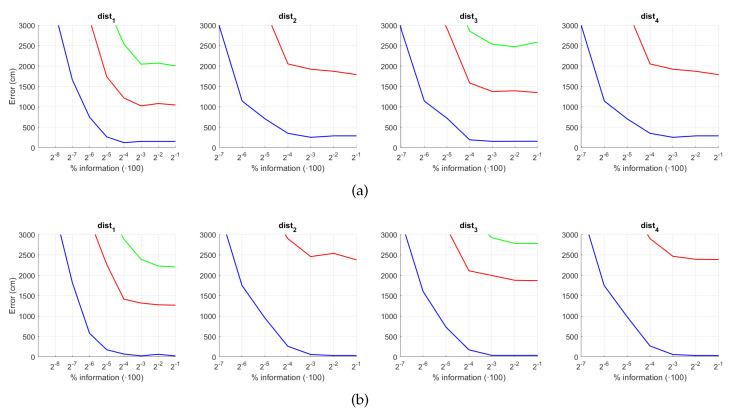
WS average localization error with noise: (**a**) no filter and (**b**) homomorphic filtering. Legend: — Original, — Noise 1, — Noise 2, — Noise 3, — Noise 4, — Noise 5.

**Figure 27:**
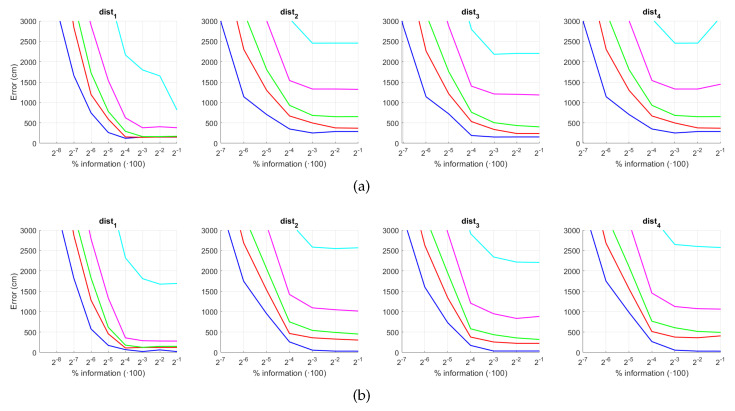
WS average localization error with occlusions: (**a**) no filter and (**b**) homomorphic filtering. Legend: — Original, — Occlusion 1, — Occlusion 2, — Occlusion 3, — Occlusion 4.

Fourthly, Figure 26 and Figure 27 present the results obtained with *Wi-SURF* considering the presence of noise or occlusions, respectively, in the test images. In these figures, first, no filter is considered (a) and second, a homomorphic filtering is carried out both to the reference and the test images (b). The information contained in each descriptor with respect to the information contained in each panoramic image can be obtained as $\left(\frac{k_{9}\cdot w_{1}\cdot 64}{N_{1}\cdot N_{2}}\cdot 100\right)$. The use of homomorphic filtering does not reduce the localization error. In this case, the performance of the descriptor is severely influenced by the presence of noise. It is very significant that results without noise and occlusion are better than the errors obtained with the previous descriptors, but when these effects appear on the scene the results worsen sharply. In general, $dist_{1}$ and $dist_{3}$ present the best results when considering an intermediate or high percentage of information.

**Figure 28:**
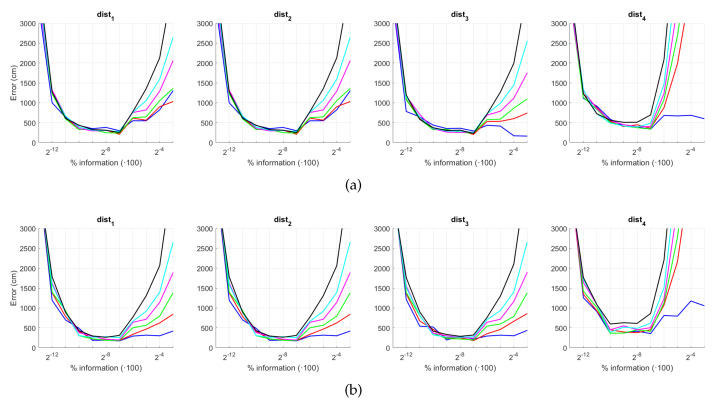
BG average localization error with noise: (**a**) no filter and (**b**) homomorphic filtering. Legend: — Original, — Noise 1, — Noise 2, — Noise 3, — Noise 4, — Noise 5.

**Figure 29:**
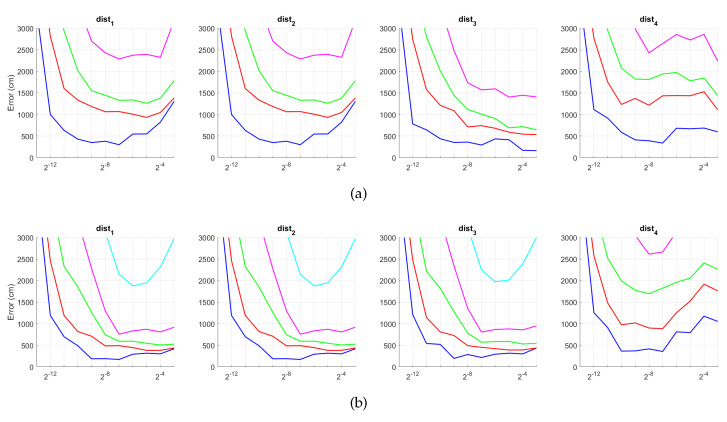
BG average localization error with occlusions: (**a**) no filter and (**b**) homomorphic filtering. Legend: — Original, — Occlusion 1, — Occlusion 2, — Occlusion 3, — Occlusion 4.

Figure 28 and Figure 29 present the results obtained with the *BRIEF-gist* method considering the presence of noise or occlusions, respectively, in the test images. In these figures, first, no filter is considered (a) and second, a homomorphic filtering is carried out both to the reference and the test images (b). Like in previous figures, the percentages in the horizontal axis express the information contained in each descriptor with respect to the information contained in each panoramic image. In the case of BG, they can be obtained as $\left(\frac{k_{10}\cdot w_{2}}{N_{1}\cdot N_{2}}\cdot 100\right)$. In this case, the best results are achieved with an intermediate amount of information, so it is not necessary to store a big quantity of information when building the descriptors. In addition, in general terms, the filter tends to improve the results. Comparing to the other description methods, *BRIEF-gist* presents higher error in ideal conditions, but it controls its error when noise appears on the scenes, obtaining good results even with high quantity of noise. Additionally it performs correctly when no occlusions take part on the image but it works wrongly when this phenomenon appears.

If we analyze jointly these results, we can arrive to some general conclusions. First, HOG presents very good localization results under ideal conditions. These results degrade in the presence of noise or occlusions, but some configurations resist these effects. Second, gist with no filter leads to worse results in ideal conditions, but it is robust against adverse effects, mainly against noise. Third, WS along with filter provides the best absolute localization results in ideal conditions. However, its performance sharply worsens with noise and occlusions. Finally, the results of BG in ideal conditions are not remarkable. However, this is the descriptor that presents more robustness in the presence of noise and occlusions, even in very unfavorable conditions.

### 5.3. Estimation of the Orientation

In this section, the problem of orientation estimation is addressed. To assess the performance of each description method in this task, independently of the results of the position estimation, the test image orientation descriptor is always compared with the orientation descriptor of the map image which was captured in the geometrically closest position. The problem is solved using the algorithms presented in Section 3, except those based on Radon transform, which proved to perform poorly in the image retrieval task.

**Figure 30:**
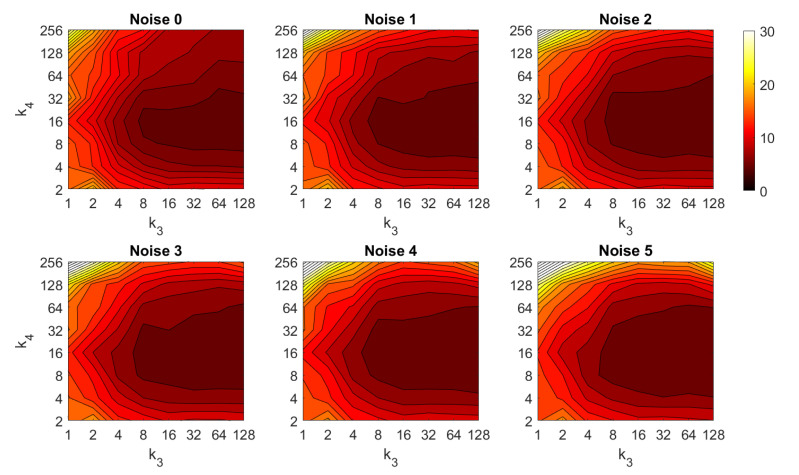
FS orientation estimation in the presence of noise. Average orientation error (deg).

**Figure 31:**
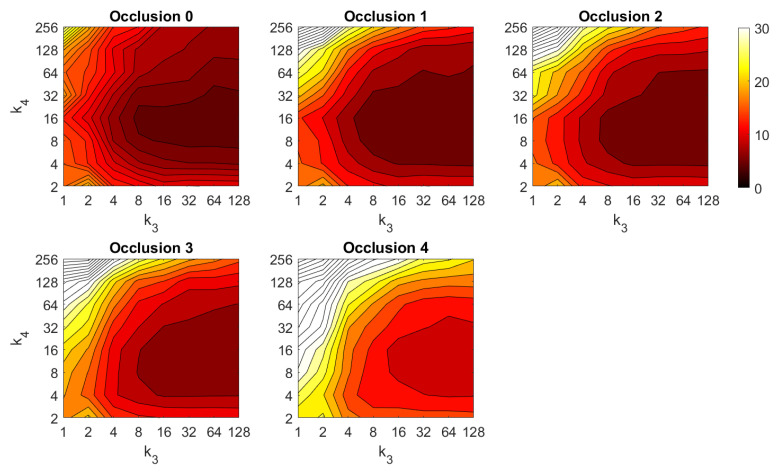
FS orientation estimation in the presence of occlusions. Average orientation error (deg).

**Figure 32:**
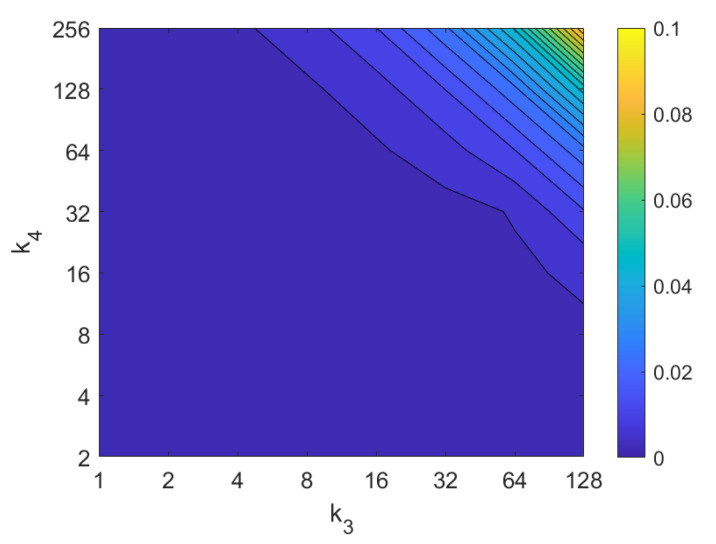
FS orientation estimation. Average computation time (s).

First, the results obtained with the Fourier Signature are presented. Figure 30 shows the results of the orientation estimation. The influence of noise is also assessed in this figure. The results are expressed as average orientation error, in degrees, after repeating the experiment with the 1232 test images. This figure shows that the algorithm is very robust against the presence of noise. The optimal configuration is an intermediate to high number of rows ($k_{3}$) and an intermediate number of columns ($k_{4}$). A high number of columns worsens the results. Additionally, the presence of occlusions in the orientation estimation process is assessed in Figure 31. This figure shows that the influence of occlusions is higher, since the results tend to worsen as the level of occlusion increases. Nevertheless, some configurations of the parameters permit obtaining an average error lower than 10 deg even with 40% occlusions. The computational time of the orientation estimation process is shown in Figure 32, expressed in seconds. The descriptor based on FS is able to estimate the orientation relatively quickly for most configurations of $k_{3}$ and $k_{4}$ and only high values of both parameters produce a relatively high computation time.

**Figure 33:**
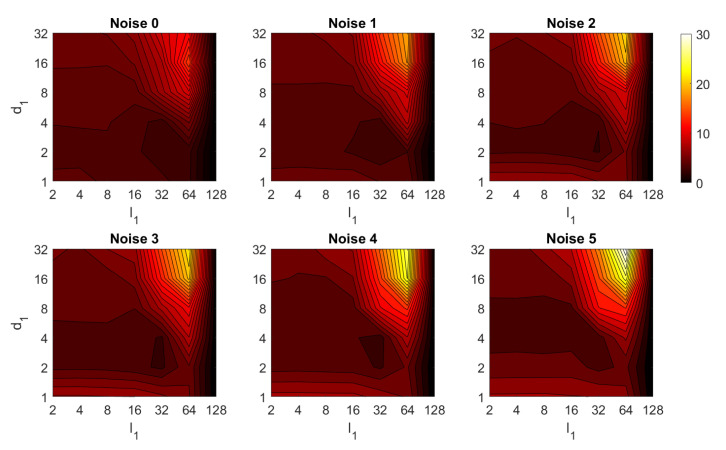
HOG orientation estimation in the presence of noise. Average orientation error (deg).

**Figure 34:**
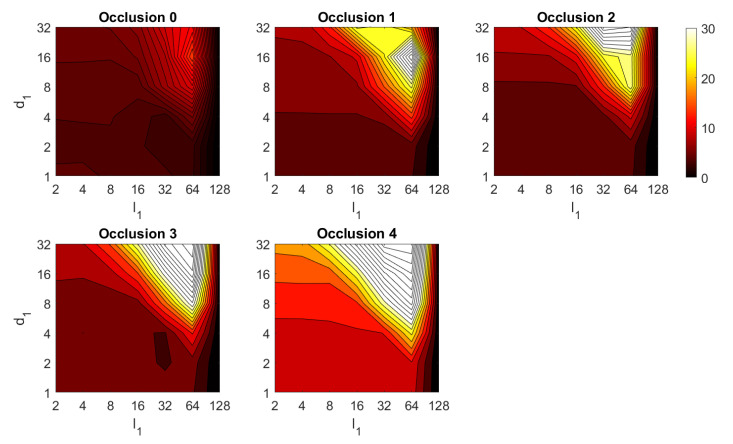
HOG orientation estimation in the presence of occlusion. Average orientation error (deg).

**Figure 35:**
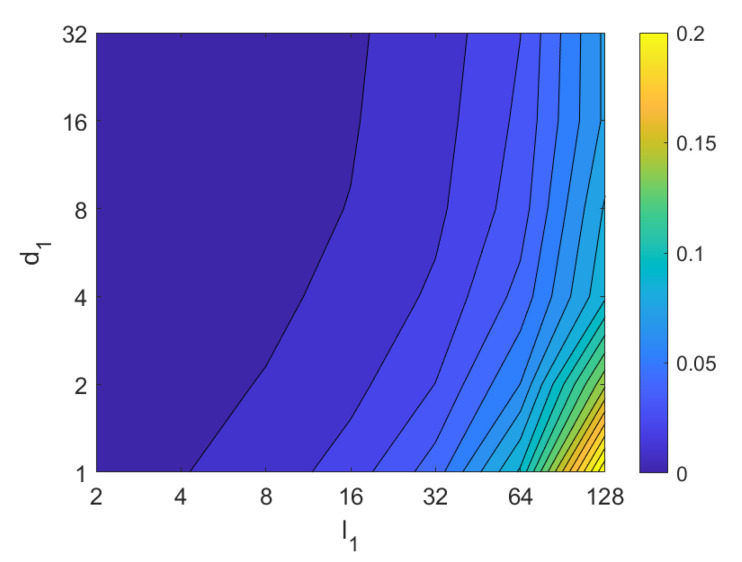
HOG orientation estimation. Average computation time (s).

Second, the performance of the HOG descriptor is assessed, considering several values of the parameters $l_{1}$ (width of the vertical cells in the orientation descriptor) and $d_{1}$ (distance between consecutive vertical cells, which are overlapped). Figure 33 shows the average orientation error after considering all the test images. In addition, the influence of the presence of different levels of noise in the test images is analyzed. In general terms, low to intermediate values of $d_{1}$ and high values of $l_{1}$ produce the best results (lower orientation error). In addition, HOG proves to be a descriptor which is robust against the presence of noise, since the results do not change substantially as the level of noise increases. In general, HOG tends to present better results in orientation estimation comparing with FS. Furthermore, the influence of partial occlusions in orientation estimation is shown in Figure 34. As with FS, the influence of occlusions in the orientation estimation is substantial, and the results degrade quickly as the percentage of occlusions increases. Notwithstanding that, high values of $l_{1}$ tend to produce relatively low orientation error, independently of the level of occlusions. Finally, Figure 35 shows the necessary time to estimate the orientation (average time, expressed in seconds, after considering all the test images). Most configurations of $l_{1}$ and $d_{1}$ produce a relatively low computation time. Only very high values of $l_{1}$ combined with low values of $d_{1}$ output a substantially high calculation time.

**Figure 36:**
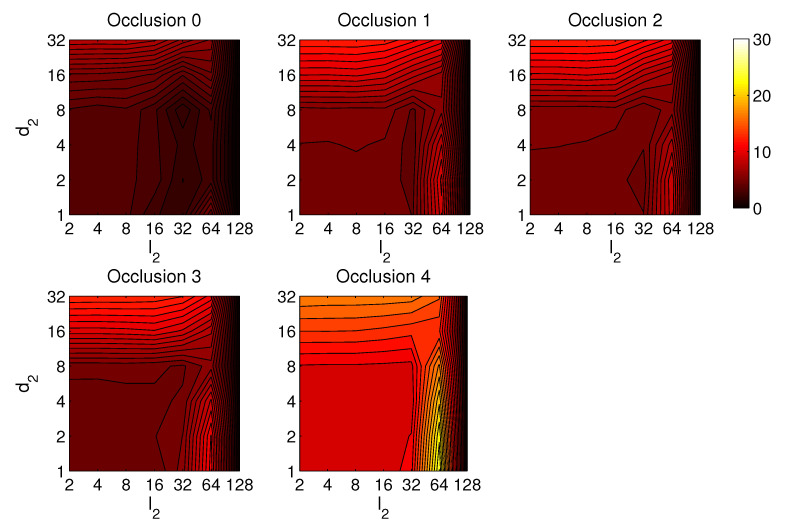
Gist orientation estimation in the presence of occlusion. Average orientation error (deg).

**Figure 37:**
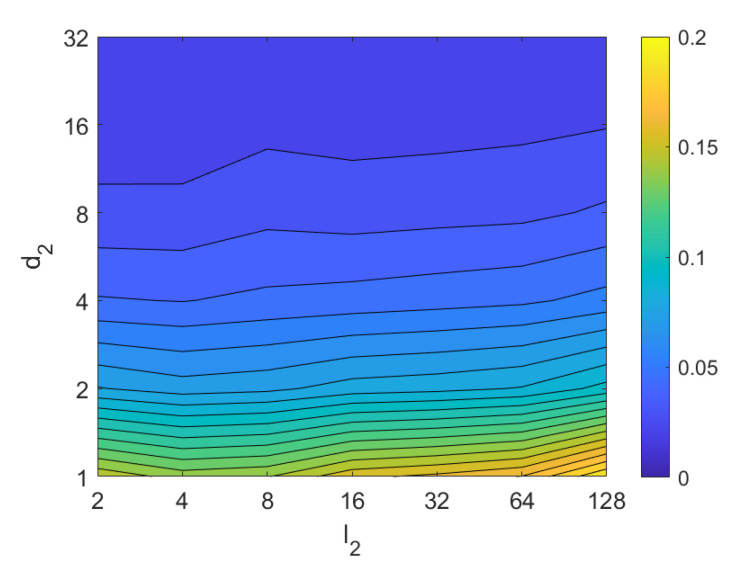
Gist orientation estimation. Average computation time (s).

Third, the results of relative orientation estimation with *gist* are presented and commented. Figure 36 shows the average orientation error (degrees) when considering different configurations of $l_{2}$ (width of the vertical blocks in the orientation descriptor) and $d_{2}$ (distance between two consecutive vertical blocks, which are overlapped). The influence of the level of occlusions is checked in this figure. In the case of this description method, the orientation error tends to increase as $d_{2}$ does. However, as in the case of HOG, high values of the width of the vertical blocks produce relatively good results independently of the value of $d_{2}$. To finish the experiments, the necessary time to estimate the orientation (average time in seconds) is shown in Figure 37. The figure shows that $d_{2}$ is the parameter that has a predominant influence upon the calculation time. Low values of this parameter produce a comparatively high computation time.

**Figure 38:**
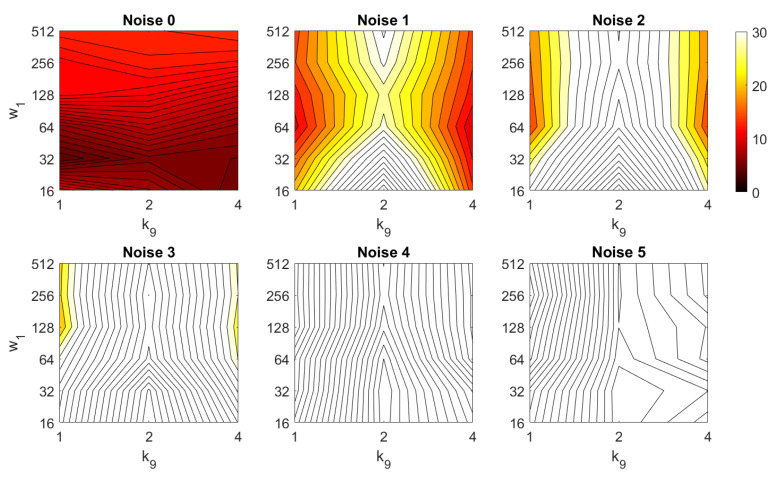
WS orientation estimation in the presence of noise. Average orientation error (deg).

**Figure 39:**
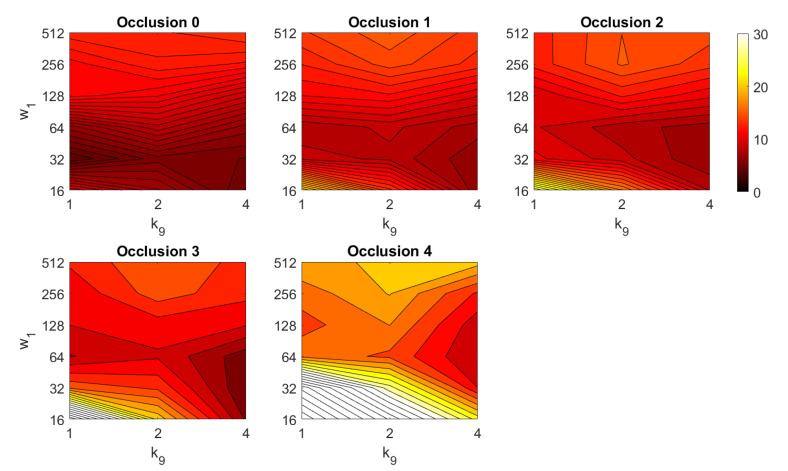
WS orientation estimation in the presence of occlusion. Average orientation error (deg).

**Figure 40:**
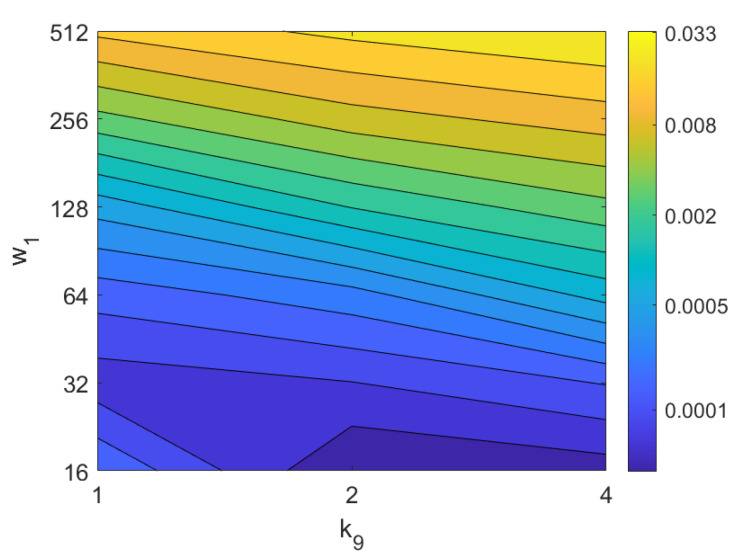
WS orientation estimation. Average computation time (s).

In addition, the results of relative orientation estimation with *Wi-SURF* are presented and commented. Figure 38 shows the average orientation error (degrees) taking into account the noise influence considering the variation on the parameters $k_{9}$ and $w_{1}$. It shows a strong influence of the noise in the result. It is possible to check that results without noise are acceptable (about 5–10 deg), but the error increase considerably when the noise appears on the scenes. If the image is corrupted with noise with variance higher than $\sigma^{2}=0.0025$, the error is always more than 30 deg. The influence of the level of occlusions can be checked in the Figure 39. In the case of the occlusions, the results show more robustness, except for the results with 40% of occlusion that are considerably bad comparing with HOG. In general, the error tends to be optimized with middle values of $w_{1}$. To finish the experiments, the necessary time to estimate the orientation (average time in seconds) is shown in Figure 40. The figure shows that $w_{1}$ is the parameter that has a predominant influence upon the calculation time. High values of this parameter produce a comparatively high computation time.

**Figure 41:**
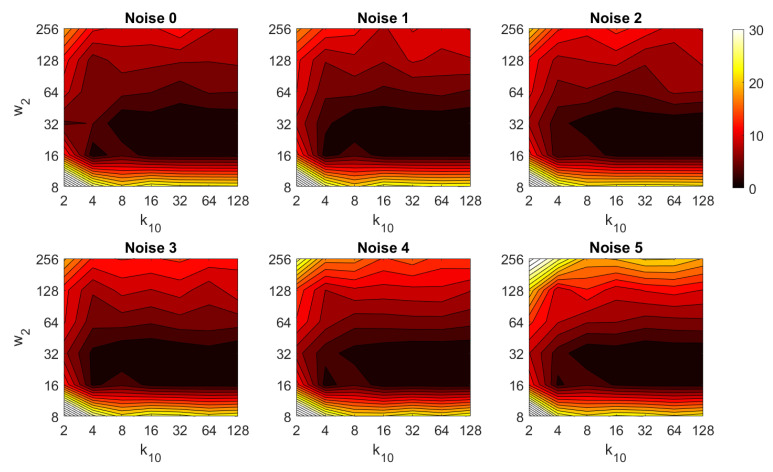
BG orientation estimation in the presence of noise. Average orientation error (deg).

**Figure 42:**
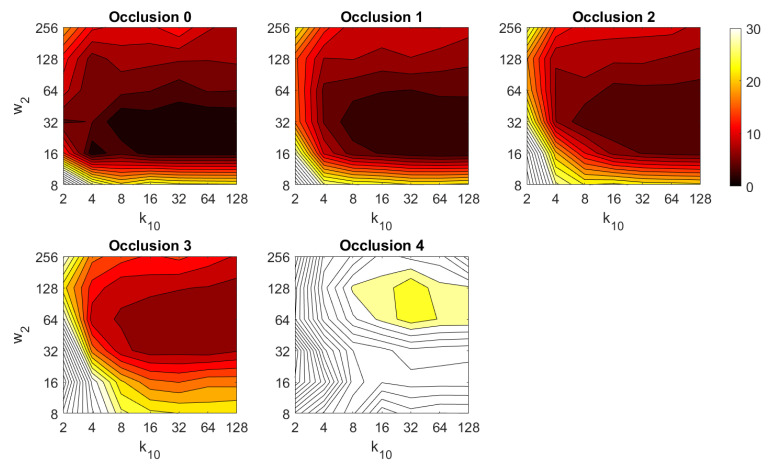
BG orientation estimation in the presence of occlusion. Average orientation error (deg).

**Figure 43:**
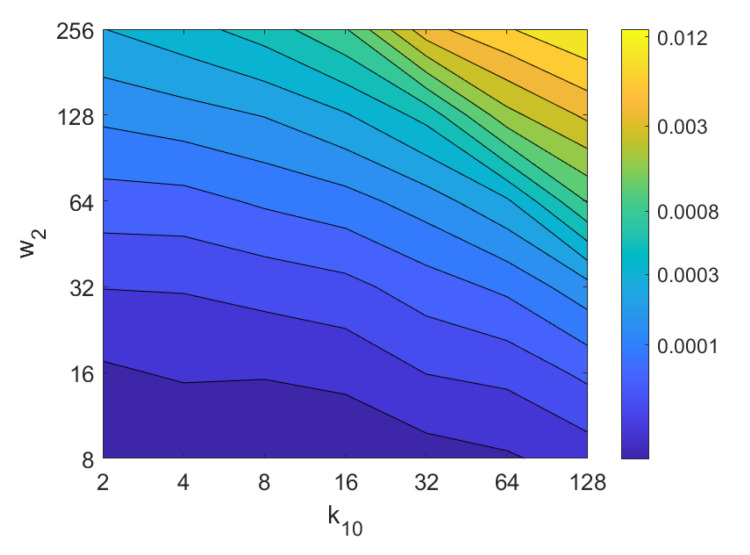
BG orientation estimation. Average computation time (s).

Additionally, the performance of the *BRIEF-gist* descriptor is assessed, considering several values of the parameters $w_{2}$ and $k_{10}$. Figure 41 shows the average orientation error after considering all the test images and the influence of the presence of different levels of noise. In general terms, the optimal configuration is an intermediate to high number of cells ($k_{10}$) and an intermediate number of windows ($w_{2}$). A high number of windows lead to worse results. In addition, *BRIEF-gist* proves to be a descriptor which is robust against the presence of noise, since the results do not change substantially as the level of noise increases. In general, *BRIEF-gist* tends to present better results in orientation estimation comparing with other descriptors. However, the influence of partial occlusions in orientation estimation has a worse influence, as shown in Figure 42. As with *Wi-SURF*, the algorithm performs considerably bad under the influence of occlusions. As before, intermediate values of $w_{2}$ output the best results. Finally, Figure 43 shows the necessary time to estimate the orientation. Most configurations of $w_{2}$ and $k_{10}$ produce a relatively low computation time. Only very high values output a substantially high calculation time.

In general terms, HOG and *gist* produce relatively better results in the estimation of relative orientation, and the previous figures prove that it is possible to find some configurations of the most relevant parameters that offer a good balance between error and calculation time. Moreover, *Wi-SURF* and *BRIEF-gist* also offer acceptable errors in ideal conditions, and the calculation times are low. However, with these two descriptors, the orientation error tends to increase remarkably with the presence of occlusions and noise.

### 5.4. Evaluation with a Trajectory Dataset

To conclude the experimental section, a new experiment is carried out with a set of images extracted from the COLD dataset [72]. This publicly available dataset contains several sets of images that were captured while a mobile robot traversed a trajectory in some indoor environments. Therefore, the results in this section permit assessing the performance of the descriptors in a different environment and with a trajectory-like dataset.

To carry out the experiment, the Saarbrücken dataset is selected [72]. To create the training set, we have selected images from the Saarbrücken dataset in such a way that the distance between consecutive capture points is, on average, 30 cm. The rest of images are considered as test images, and they are used to solve the localization problem, as described in Section 3.

**Figure 44:**
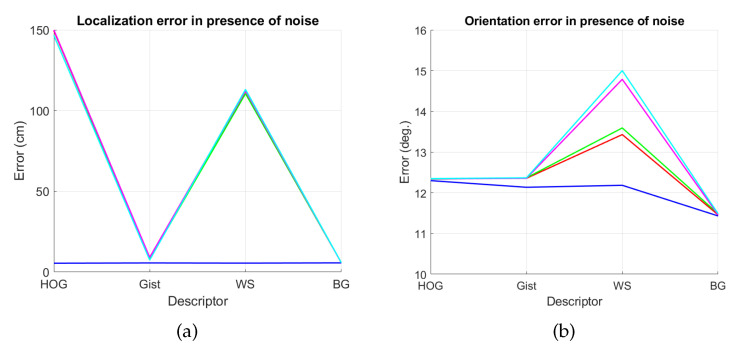
Average errors with the COLD dataset in the presence of noise. (**a**) Average position error (cm) and (**b**) average orientation error (deg). Legend: — Original, — Noise 1, — Noise 2, — Noise 3, — Noise 4.

**Figure 45:**
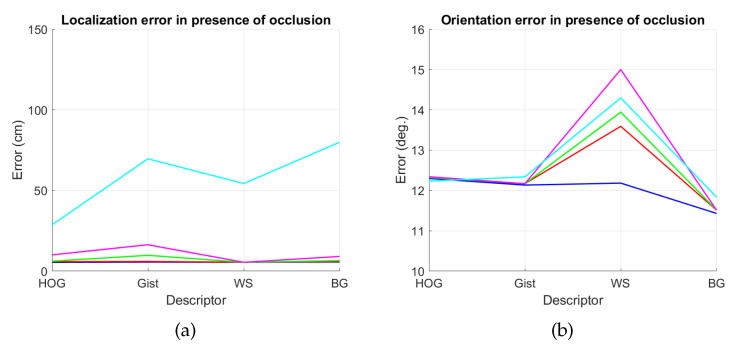
Average errors with the COLD dataset in the presence of occlusions. (**a**) Average position error (cm) and (**b**) average orientation error (deg). Legend: — Original, — Occlusion 1, — Occlusion 2, — Occlusion 3, — Occlusion 4.

The results are presented in Figure 44 and Figure 45. As in the previous experiments, we estimate both the position and the relative orientation of the robot and we consider either noise or occlusions in the test images. The descriptors included in this experiment are HOG, *gist*, WS and BG, since they have showed a good performance in the previous experiments. Additionally, their most relevant parameters are tuned with the values that provided, in general, best estimations in the previous subsections. The levels of noise or occlusion are the same than those included in the previous experiments: presence of different Gaussian noise ($\sigma^{2}=\left\{0,0.0025,0.05,0.01,0.02\right\}$) and partial occlusions considering ({0,5,10,20,40}%) of the image occluded.

First, Figure 44 shows (a) the average error of the localization task (expressed in cm) and (b) the average error of the orientation retrieval task (expressed in deg). Several levels of noise are considered in this experiment. Second, Figure 45 shows the same results but considering several levels of partial occlusions. It is worth highlighting that these errors cannot be directly compared with the absolute errors presented in the previous subsections, since the experimental setup is different. Notwithstanding that, these figures permit assessing the relative performance of the descriptors with a trajectory-like dataset and knowing if the descriptors present similar tendencies in different kinds of environments and datasets.

Figure 44a shows that the relative performance of the descriptors when calculating the relative position in ideal conditions (i.e., with no noise) is quite similar. Additionally, *gist* and BG resist quite well the presence of noise. However, HOG and WS quickly degrade their performance as the level of noise increases. These results are in line with those presented in previous sections. About the relative orientation retrieval with noise, Figure 44b shows that HOG, *gist* and BG are quite robust, while WS performs worse with high levels of noise. Figure 45a proves that the four description methods present relatively good results in the presence of occlusions, except for the highest level of occlusion. In this case, HOG is the descriptor that best performs. About the orientation retrieval in presence of occlusions, Figure 45b shows that HOG, *gist* and BG perform well, independently on the level of occlusion, but WS quickly increases the error with high levels of occlusion.

## 6. Conclusions

This paper has focused on the study of the localization problem, using a previously built visual representation of the environment. The problem has been addressed as an absolute localization task, making use of the data provided by a catadioptric vision sensor mounted on the robot both to estimate both the position and the orientation of the robot. To extract relevant information from the images, methods based on the global appearance of the panoramic scenes have been implemented and assessed. A comparative evaluation has been carried out between six families of well-known global description methods.

The main contributions of the paper include an exhaustive study of global appearance techniques (FS, HOG, *gist*, WS, BG and RT) and the adaptation of some of these algorithms to store position and orientation information from panoramic scenes in such a way that both processes can be carried out sequentially. First, the position of the robot can be estimated and second, the orientation is estimated.

In addition, the computational cost to estimate the position and orientation has been studied, including the influence of the most relevant parameters. This study has revealed that FS and RT present a reasonable computational cost, and so do some specific configurations of HOG and *gist*, but *Wi-SURF* and *BRIEF-gist* are less competitive as far as computation time is concerned. From this point of view, FS, RT, HOG and *gist* could be feasible in real time applications. In addition to this, the performance of the descriptors has been tested in localization tasks. First, we have focused on the image retrieval problem. All the description methods have been tested along with several distance measures, and the results have shown that *Wi-SURF* and *BRIEF-gist* present the best relative results. Additionally, HOG with certain distance measures present very good results and the best relation between computational time and image retrieval rate. Second, the relative error of the position estimation has been studied. It has corroborated that: (a) HOG presents very good localization results under ideal conditions and is quite robust to noise and occlusions, (b) Wi-SURF provides the most competitive results under ideal conditions but is very negatively influenced by noise and occlusions and (c) BRIEF-gist is very robust against these effects, but its results in ideal conditions are not remarkable. To finish, the problem of orientation estimation has been addressed. The best results are obtained with WS and BG but only when there is neither noise nor occlusions. If these phenomena are present, HOG and *gist* perform more robustly.

These results have demonstrated that global-appearance methods are a feasible approach to solve the localization task. Thanks to them, the robot can build a model of the environment and use it to estimate with accuracy the position and orientation of the robot in the environment, with computational efficiency. This fact may have interesting implications in future developments in the field of mobile robotics. As an example, this concept can be used to build hybrid maps that arrange the information in several layers, with different accuracy: a high level layer that permits carrying out a rough and quick localization and a lower layer that contains information with geometric accuracy and allows the robot to refine the estimation of its position. Global-appearance methods can be used on their own or in conjunction with feature-based techniques to develop algorithms that face these problems efficiently.

All these facts encourage us to go into this framework in depth. To build a fully autonomous mapping and localization system, several future works should be considered. First, the image collection process could be automated to obtain an optimal representation of the environment. Second, the mapping and localization processes could be integrated in a topological SLAM system that carries out both the model creation and the localization from the scratch. To optimize these algorithms we also consider carrying out a complete comparison between global-appearance and feature-based techniques as a future work.

## Figures and Tables

**Table 1:** Parameters whose influence in the localization process is studied.

Descriptor	Parameters
*FS*	$k_{1}\Rightarrow$ number of rows, position descriptor $A_{j}$ $k_{2}\Rightarrow$ number of columns, position descriptor $A_{j}$ $k_{3}\Rightarrow$ number of rows, orientation descriptor $\Phi_{j}$ $k_{4}\Rightarrow$ number of columns, orientation descriptor $\Phi_{j}$
*HOG*	$b_{1}\Rightarrow$ number of bins per histogram, position descriptor ${\vec{h}}_{1j}$ $k_{5}\Rightarrow$ number of horizontal cells, position descriptor ${\vec{h}}_{1j}$ $b_{2}\Rightarrow$ number of bins per histogram, orientation descriptor ${\vec{h}}_{2j}$ $l_{1}\Rightarrow$ width of vertical cells, orientation descriptor ${\vec{h}}_{2j}$ $d_{1}\Rightarrow$ distance between vertical cells, orientation descriptor ${\vec{h}}_{2j}$ $k_{6}=\frac{N_{2}}{d_{1}}\Rightarrow$ number of vertical cells, orientation descriptor ${\vec{h}}_{2j}$
*Gist*	$m_{1}\Rightarrow$ number of orientations (Gabor filters), position descriptor ${\vec{g}}_{1j}$ $k_{7}\Rightarrow$ number of horizontal blocks, position descriptor ${\vec{g}}_{1j}$ $m_{2}\Rightarrow$ number of orientations (Gabor filters), orientation descriptor ${\vec{g}}_{2j}$ $l_{2}\Rightarrow$ width of vertical blocks, orientation descriptor ${\vec{g}}_{2j}$ $d_{2}\Rightarrow$ distance between vertical blocks, orientation descriptor ${\vec{g}}_{2}$ $k_{8}=\frac{N_{2}}{d_{2}}\Rightarrow$ number of vertical blocks, orientation descriptor ${\vec{g}}_{2j}$
*WS*	$w_{1}\Rightarrow$ number of windows per cell, descriptor ${\vec{ws}}_{j}$ $k_{9}\Rightarrow$ number of horizontal blocks, descriptor ${\vec{ws}}_{j}$ $sp_{1}\Rightarrow$ horizontal space between windows, descriptor ${\vec{ws}}_{j}$
*BG*	$w_{2}\Rightarrow$ number of windows per cell, descriptor ${\vec{bg}}_{j}$ $k_{10}\Rightarrow$ number of horizontal blocks, descriptor ${\vec{bg}}_{j}$
*RT*	$p_{1}\Rightarrow$ degrees between lines where Radon is calculated, matrix *r* $k_{11}\Rightarrow$ number of columns, position descriptor ${A_{\mathrm{RT}}}_{j}$ $k_{12}\Rightarrow$ number of columns, orientation descriptor ${\Phi_{RT}}_{j}$ $N_{x}\Rightarrow$ omnidirectional images’ size is $N_{x}\times N_{x}$

**Table 2:** Contents of the map, for localization and orientation estimation, per image included in the model $im_{j},\;j=1,\cdots ,n$.

Descriptor	Localization	Orientation
*FS*	$A_{j}\in R^{k_{1}\times k_{2}}$	$\Phi_{j}\in R^{k_{3}\times k_{4}}$
*HOG*	${\vec{h}}_{1j}\in R^{k_{5}\cdot b_{1}\times 1}$	${\vec{h}}_{2j}\in R^{k_{6}\cdot b_{2}\times 1}$
*Gist*	${\vec{g}}_{1j}\in R^{2\cdot k_{7}\cdot m_{1}\times 1}$	${\vec{g}}_{2j}\in R^{k_{8}\cdot m_{2}\times 1}$
*WS*	${\vec{ws}}_{j}\in R^{k_{9}\cdot w_{1}\cdot 64\times 1}$	
*BG*	${\vec{bg}}_{j}\in R^{k_{10}\cdot w_{2}\times 1}$	
*RT–F*	${A_{\mathrm{RT}}}_{j}\in R^{\frac{360}{p_{1}}\times k_{11}}$	${\Phi_{RT}}_{j}\in R^{\frac{360}{p_{1}}\times k_{12}}$
*RT–POC*	$r\in R^{\frac{360}{p_{1}}\times 0.5\cdot N_{x}}$	

## Data Availability

Publicly available datasets were analyzed in this study. This data can be found here: http://arvc.umh.es/db/images/quorumv/ (accessed on 9 May 2021).
